# Live imaging of late-stage preimplantation human embryos reveals de novo mitotic errors

**DOI:** 10.1038/s41587-025-02851-1

**Published:** 2025-10-23

**Authors:** Ahmed Abdelbaki, Afshan McCarthy, Anita Karsa, Leila Muresan, Kay Elder, Athanasios Papathanasiou, Phil Snell, Leila Christie, Martin Wilding, Benjamin J. Steventon, Kathy K. Niakan

**Affiliations:** 1 The Loke Centre for Trophoblast Research, Department of Physiology, Development and Neuroscience, University of Cambridge, Cambridge, UK; 2 Human Embryo and Stem Cell Laboratory, The Francis Crick Institute, London, UK; 3 Department of Zoology, Faculty of Science, Zagazig University, Zagazig, Egypt; 4 Cambridge Advanced Imaging Centre and Department of Physiology, Development and Neuroscience, University of Cambridge, Cambridge, UK; 5 School of Computing and Information Science, Anglia Ruskin University, Cambridge, UK; 6 Bourn Hall Clinic, Bourn, Cambridge, UK; 7 Create Fertility, London, UK; 8 Department of Genetics, University of Cambridge, Cambridge, UK; 9 Wellcome–MRC Cambridge Stem Cell Institute, University of Cambridge, Cambridge, UK; 10 Epigenetics Programme, Babraham Institute, Cambridge, UK

**Keywords:** Time-lapse imaging, Chromosome segregation, Image processing

## Abstract

Existing methods to image chromosome segregation errors are not suitable for studying human embryos at advanced preimplantation stages. As chromosomal errors are a leading cause of miscarriage and infertility, it remains unclear whether missegregation arises postfertilization. Here we optimize nuclear DNA labeling via messenger RNA electroporation and apply light-sheet live imaging to reveal chromosome segregation errors immediately before implantation. We show that embryos at advanced preimplantation stages display missegregation, including multipolar spindle formation, lagging chromosomes, misalignment and mitotic slippage. Most lagging chromosomes are passively inherited rather than reincorporated. To trace individual nuclei, we developed an open-source, semi-automated segmentation method using a customized deep learning model optimized for variability in embryo size, shape and signal. With this approach, we find most labeled cells remain externally positioned, consistent with placental rather than inner cell mass fate. Our findings raise questions about clinical uses of preimplantation genetic testing for aneuploidy, while providing broadly applicable imaging and segmentation methods for studying diverse cellular structures in human embryos.

## Abstract (web summary)

A method to label cultured human embryos finds chromosome segregation errors in placenta-fated cells.

## Main

Following fertilization, human zygotes undergo a series of cell divisions, forming a blastocyst before implantation. The majority of human blastocysts display a mix of both normal euploid and abnormal aneuploid cells^1–4^. In contrast to aneuploidies of meiotic origin, which can affect all daughter cells, mosaic aneuploidy is more likely due to mitotic errors after fertilization^3,4^. Aneuploidy is known to contribute to implantation failure and embryonic arrest^5–7^, although mosaic aneuploidy is thought to be tolerated especially in placental-fated trophectoderm cells^8–10^.

Numerous studies have indicated that mosaicism frequently arises in 30–70% of cleavage-stage embryos^4,11^ and approximately 10–30% of blastocysts^12–15^. Evidence for this comes from observation of spindle and nuclear abnormalities in fixed human embryos at static time points. Although mitotic errors in cleavage-stage embryos have been described, mitotic errors in chromosome segregation arising de novo at the blastocyst stage in human preimplantation embryos are less well understood.

Characterization of these mitotic errors at later stages in human embryos has been limited by challenges in nuclear labeling, live-cell imaging and tracking. In recent years, advances in live imaging of mouse embryos have expanded our knowledge of chromosome segregation as well as cell fate specification in early mammalian development^16–18^. There is comparatively limited understanding of cell divisions and timing of cell fate specification in human blastocysts.

Recent reports have achieved labeling of human embryos through microinjection of mRNA in zygotes^19^ or by using live DNA dyes^17^ and reported chromosome segregation errors including micronuclei formation and nuclear shedding, respectively. However, microinjections are not suitable for most human embryos donated for research, as these are often obtained at the blastocyst stage with over 100 cells. Prolonged incubation with live DNA dyes has been shown to induce DNA damage responses and directly impact mitotic progression^18,20^. Furthermore, all previous studies imaged human embryos using confocal microscopy, which is not suitable for long-term imaging due to high phototoxicity^21^. Light-sheet fluorescence microscopy offers an important improvement in illumination and detection, which minimizes the extent of light exposure and enables long-term imaging^21,22^.

Here we systematically tested various nuclear labeling methods and developed an electroporation method to introduce mRNA in human embryos at the blastocyst stage. We used live imaging by light-sheet microscopy to demonstrate that chromosome segregation errors arise in human blastocysts, including de novo multipolar divisions, lagging chromosomes, misalignment and chromosome slippage. By tracking lagging chromosomes after mitosis, we observed that in some cells, micronuclei were passively inherited by one daughter cell without reincorporation in subsequent mitoses, whereas in other cells there was evidence of reincorporation. We developed a semi-automated pipeline to track the position of labeled cells at the blastocyst stage and found that although most cells remained within the placental progenitor compartment, a rare contribution of an individual cell to the inner cells within the blastocoel cavity was observed. Altogether, we optimized labeling, live imaging and tracking methods to characterize previously challenging stages of human development, thereby making the discovery of de novo mitotic errors at a late stage just before implantation.

## Results

### Optimized strategies for nuclear DNA labeling in mouse and human embryos

To track and trace nuclei during live imaging of human preimplantation development, we initially systematically investigated various labeling methods using mouse embryos. We required a method that allowed us to label late-stage preimplantation embryos with high efficiency and to track cells for 48 h without negatively influencing cell proliferation or development. We compared fluorescent labeling methods using lentivirus, adeno-associated virus (AAV), baculovirus (BacMam), DNA dyes and electroporation of mRNAs.

**Extended Data Fig. 1:**
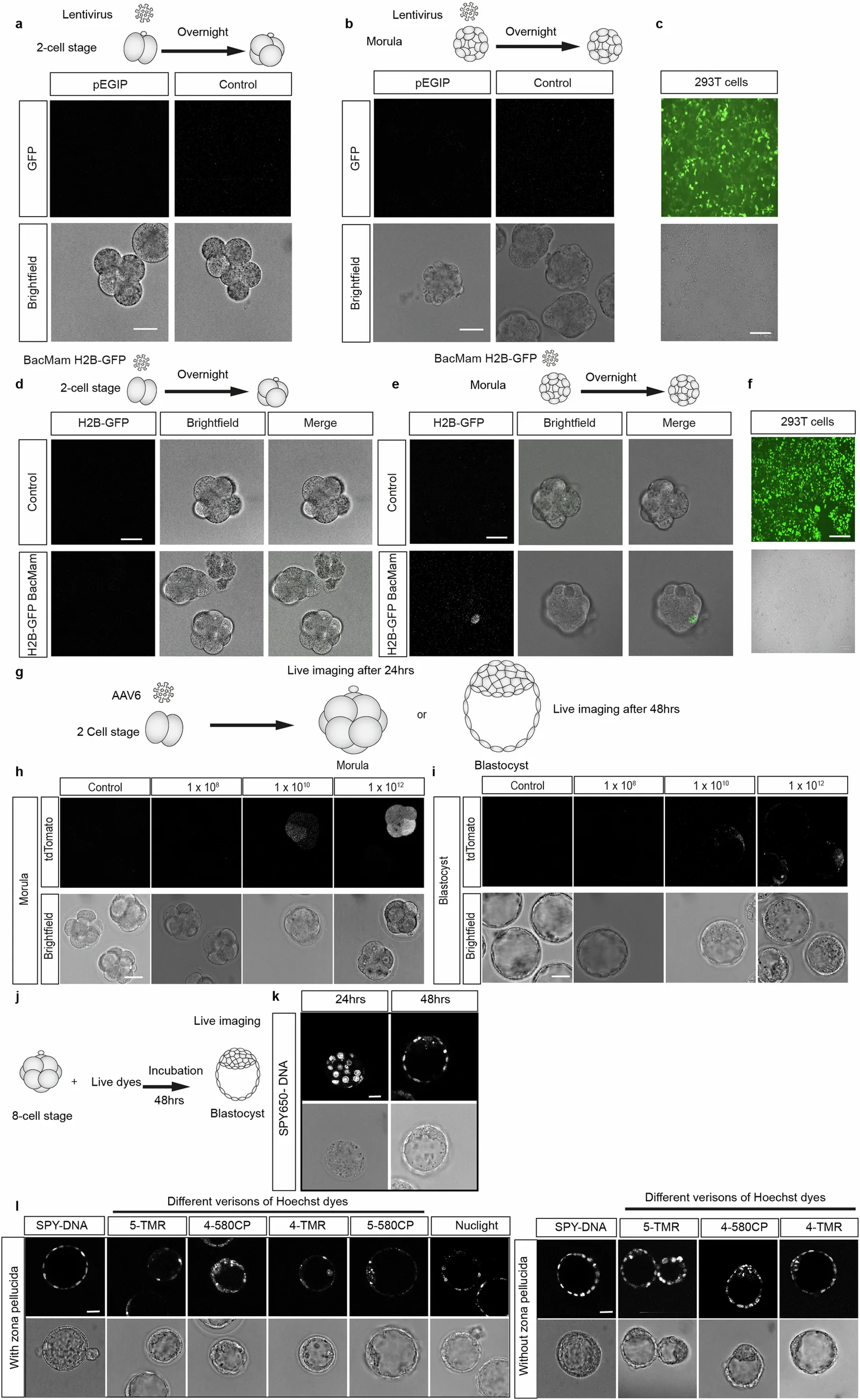
Viral vector-mediated transduction and live DNA staining approaches in early mouse embryos and HEK 293 T cells. **a,b** Representative images of mouse embryos transduced with a pEGIP lentiviral vector that carries an EGFP gene downstream of the EF-1a promoter. Embryos were washed tree times with hyaluronidase for 10 s and cultured in global media. The zona pellucida of mouse embryos was removed prior to transduction. Mouse zona-free embryos were then divided into two groups for transduction at the: **(a)** two cell stage or at the **(b)** morula stage. Embryos were culture in Global medium containing pEGIP lentiviral vectors or in control Global medium in the absence of lentivirus. Embryos were then imaged live 24 h following transduction. **c**, Transduction of HEK 293 T cells with concentrated pEGIP lentivirus was performed as a control confirming efficacy of EGFP lentiviral vectors. **d,e** CellLight™ Histone H2B-GFP, BacMam 2.0 baculovirus transduction of early mouse embryos from the **(d)** 2-cell stage or the **(e)** morula stage. H2B-GFP BacMam transduced mouse embryos were imaged 24 hrs after transduction. **f**, Transduction of HEK 293 T cells with H2B-GFP BacMam was performed as a control confirming GFP expression. **g**, Schematic of AAV6-mediated transduction of embryos. **h,i** Mouse 2-cell stage embryos were cultured in the presence of AAV6 adeno-associated virus carrying a tdTomato gene downstream of the EF-1a promoter. The expression of tdTomato was analyzed by live cell imaging at the **(h)** morula or **(i)** blastocyst stage. Data from three independent experiments. **j**, Schematic representation of 8-cell stage mouse embryos cultured in Global medium containing live dyes for 24 or 48 hrs until the blastocyst stage. Mouse embryos were subsequently imaged on a confocal microscope in the presence of the dyes. **k**, Representative images of embryos stained with SPY650-DNA after 24 hrs and 48 hrs and phase-contrast images. Embryos were stained from the 8-cell stage until the times indicated. **l**, Representative images of embryos stained with various live DNA dyes (SPY650-DNA, 5-TMR-Hoechst, 4-TMR-Hoechst, 4-580CP-Hoechst, 5-580CP-Hoechst, and NucLight Rapid Red). Imaging was conducted 48 hrs post-staining. Phase contrast images are shown. Data from three independent experiments (**k-l**). Scale bar, 30 μm.

We initially labeled four-cell-stage mouse embryos because this allowed us to determine the perdurance of labeling for 48 h. Mouse embryos were transduced with high-titer lentivirus carrying an H2B-GFP reporter, BacMam H2B-GFP or AAV serotype 6 (AAV6)-GFP. These vectors expressed GFP under the control of a constitutive elongation factor 1a (EF1a) promoter. We monitored embryo development and fluorescence expression over time for 48 h. We showed that while lentiviral transduction in HEK 293T cells is robust, H2B-GFP expression was not detected following lentiviral transduction in any of the embryos analyzed (Extended Data Fig. 1a–c), indicating silencing. Baculovirus (BacMam) showed faint signals in one cell at the morula stage in 1 of 20 embryos imaged (Extended Data Fig. 1d–f). AAV6 exhibited transient low expression, lasting only 24 h (Extended Data Fig. 1g–i). We therefore excluded these methods from subsequent analysis in this early embryo context.

We next examined different DNA dye labeling methods in live cells including SPY650-DNA, 5-TMR-Hoechst, 4-TMR-Hoechst, 4-580CP-Hoechst, 5-580CP-Hoechst and Nuclight Rapid Red. Mouse embryos were cultured continuously in media containing different DNA dyes from the four-cell stage and imaged after 48 h. Among all the tested DNA dyes, SPY650-DNA dye stained the majority of cells at the cleavage stage. This initially looked promising; however, only nuclei of trophectoderm cells at the blastocyst stage were labeled (Extended Data Fig. 1j–l). By contrast, embryo- and yolk sac-fated cells comprising the inner cell mass exhibited nonspecific cytoplasmic staining (Extended Data Fig. 1j–l).

**Extended Data Fig. 2:**
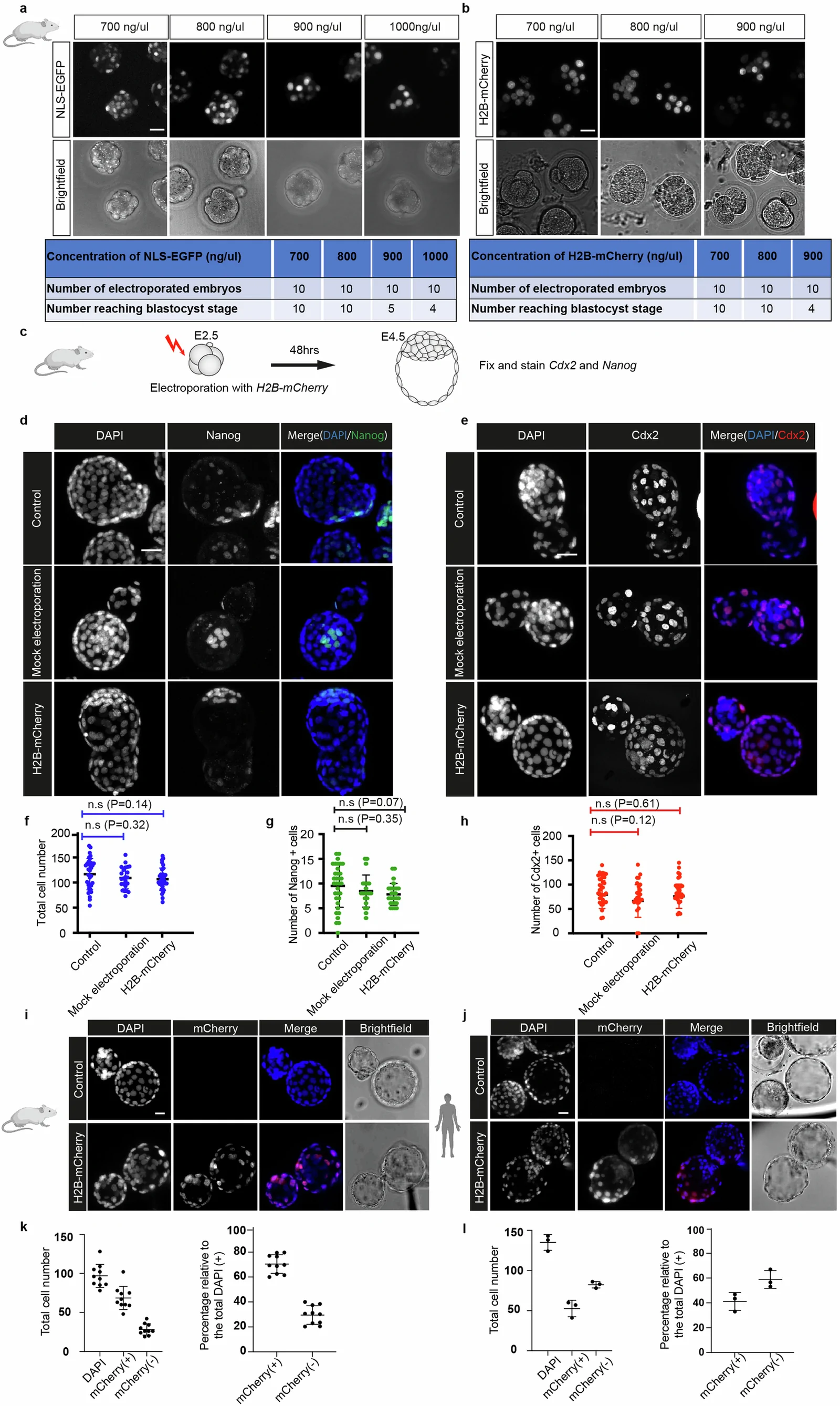
Development of mouse embryos electroporated with various concentrations of mRNA and transfection efficiency following electroporation of mouse and human embryos. **a**, Mouse embryos were electroporated at 4-cell stage with an *in vitro* transcribed EGFP mRNA containing a nuclear localisation signal (NLS) at the concentrations shown. Fluorescent and phase-contrast images were taken 24 hrs after electroporation. The number of embryos developing to the blastocyst stage is summarised. n = 10 embryos electroporated per concentration. **b**, Mouse embryos were electroporated at 4-cell stage with an *in vitro* transcribed H2B-mCherry mRNA at the concentrations shown. Fluorescent and phase-contrast images were taken 24 hrs after electroporation. The number of embryos developing to the blastocyst stage is summarised. n = 10 embryos electroporated per concentration. **c**, Schematic of the experimental setup of mouse embryos electroporated with 500 ng/µl H2B-mCherry mRNA followed by 48 hrs culture in Global media to the blastocyst stage. **d,e** Control mouse embryos cultured without electroporation (n = 33), a mock electroporation group (n = 23), and embryos electroporated with 500 ng/µl H2B mCherry mRNA (n = 32) were immunofluorescently analysed for the expression of **(d)** Nanog (epiblast molecular marker), **(e)** Cdx2 (trophectoderm molecular marker) and DAPI nuclear staining. **f**, Quantification of total cell number; **g**, Nanog-positive cells; or **h**, Cdx2-positive cells. No difference in the number of Cdx2-positive and Nanog-positive cells was observed. P > 0.05 NS, not significant, (two-tailed t-test). Scale bars, 30 μm. Error bars represent the mean ± s.d. Data from three independent experiments. Electroporation efficiency in mouse and human embryos. Embryos electroporated with H2B-mCherry were stained with DAPI (nuclear dye) at endpoint of time-lapses imaging. Individual cells were counted and categorized as DAPI-positive and mCherry-positive or -negative. **i, j** Representative images of mouse and human embryos, respectively, showing nuclear staining (DAPI) and reporter expression (H2B-mCherry). **k, l** Percentage quantification of electroporation efficiency, showing the proportion of mCherry-positive cells relative to the total DAPI-positive population. Mouse (n = 10) and human embryos (n = 3), Scale bar, 30 μm. Error bar indicates the mean ± s.d. Source data

To further explore methods for nuclear DNA labeling, we optimized mRNA electroporation of mouse cleavage-stage embryos initially using an enhanced GFP fused to a nuclear localization signal (*NLS-EGFP*) or *H2B-mCherry* mRNA. We identified electroporation parameters that allowed chromosome labeling following electroporation of mouse embryos at cleavage and blastocyst stages. We observed that electroporation of mRNA from the four-cell stage at a concentration ranging from 700 ng µl^−1^ to 800 ng µl^−1^ had no obvious impact on progression of development to the blastocyst stage (Extended Data Fig. 2a,b). We subsequently electroporated *H2B-mCherry* mRNA and quantified the expression of lineage-associated molecular markers of the trophectoderm (CDX2) and embryo-fated epiblast (NANOG) (Extended Data Fig. 2c–e). We observed no difference in total cell number or the proportion of trophectoderm or epiblast cells between electroporated and control embryos (Extended Data Fig. 2f–h). We next electroporated blastocyst-stage mouse and human embryos and assessed mRNA electroporation efficiency in each of these species. We found that the efficiency was approximately 75% and 41% in mouse and human embryos, respectively (Extended Data Fig. 2k,l). We therefore progressed with the use of mRNA electroporation for nuclear DNA labeling of mouse and human embryos before live imaging.

### Light-sheet live imaging reveals differences in interphase duration between mouse and human embryos

**Fig. 1:**
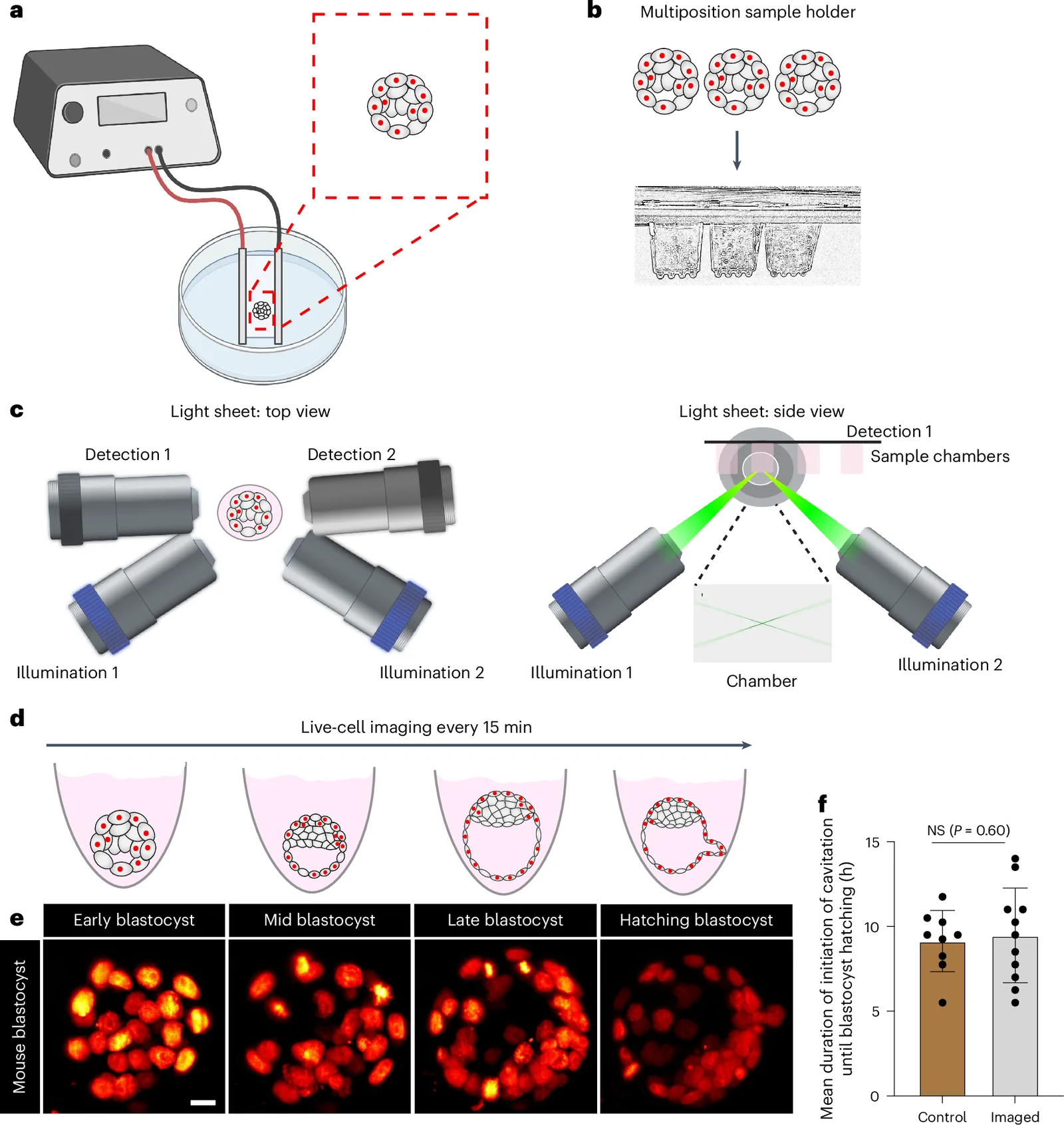
Dual-view light-sheet microscope imaging of preimplantation embryos. **a**, Schematic of the experimental setup: mouse embryos were electroporated with *H2B-mCherry* mRNA and allowed to recover for 2 h before live-embryo light-sheet imaging. **b**, Embryos were transferred to the multiposition sample holder. **c**, Schematic showing the configuration of two detection and two illumination chambers from the top and side views of the microscope sample chamber. The magnified view of the sample chamber displays the illumination beams. **d**, Mouse embryos expressing H2B-mCherry were imaged by light-sheet microscopy from the blastocyst stage at the selected stages shown. **e**, Light-sheet imaging of embryos labeled with H2B-mCherry enables visualization of mouse embryo development. Scale bar, 20 µm. **f**, The duration of initiation of cavitation until blastocyst hatching was quantified in mouse embryos labeled with H2B-mCherry and subject to live-embryo fluorescent light-sheet imaging compared with unlabeled control embryos not subject to light-sheet imaging incubated in conventional conditions. Both groups exhibit similar developmental timing (*n* = 10 embryos per group). Statistical analysis by two-tailed *t*-test. NS, not significant. Error bars represent the mean ± s.d. Illustrations in **a**, **c** and **d** created using BioRender.com. Source data

We next optimized live imaging of nuclear-labeled mouse embryos using light-sheet microscopy based on methods published previously^21^. We selected the LS2 light-sheet microscope because it has dual illumination and double detection to capture a dual view of samples (Fig. 1a–c). We confirmed that developmental timing and blastocyst progression were not significantly different between light-sheet-imaged and nonimaged control embryos (Fig. 1d–f).

**Fig. 2:**
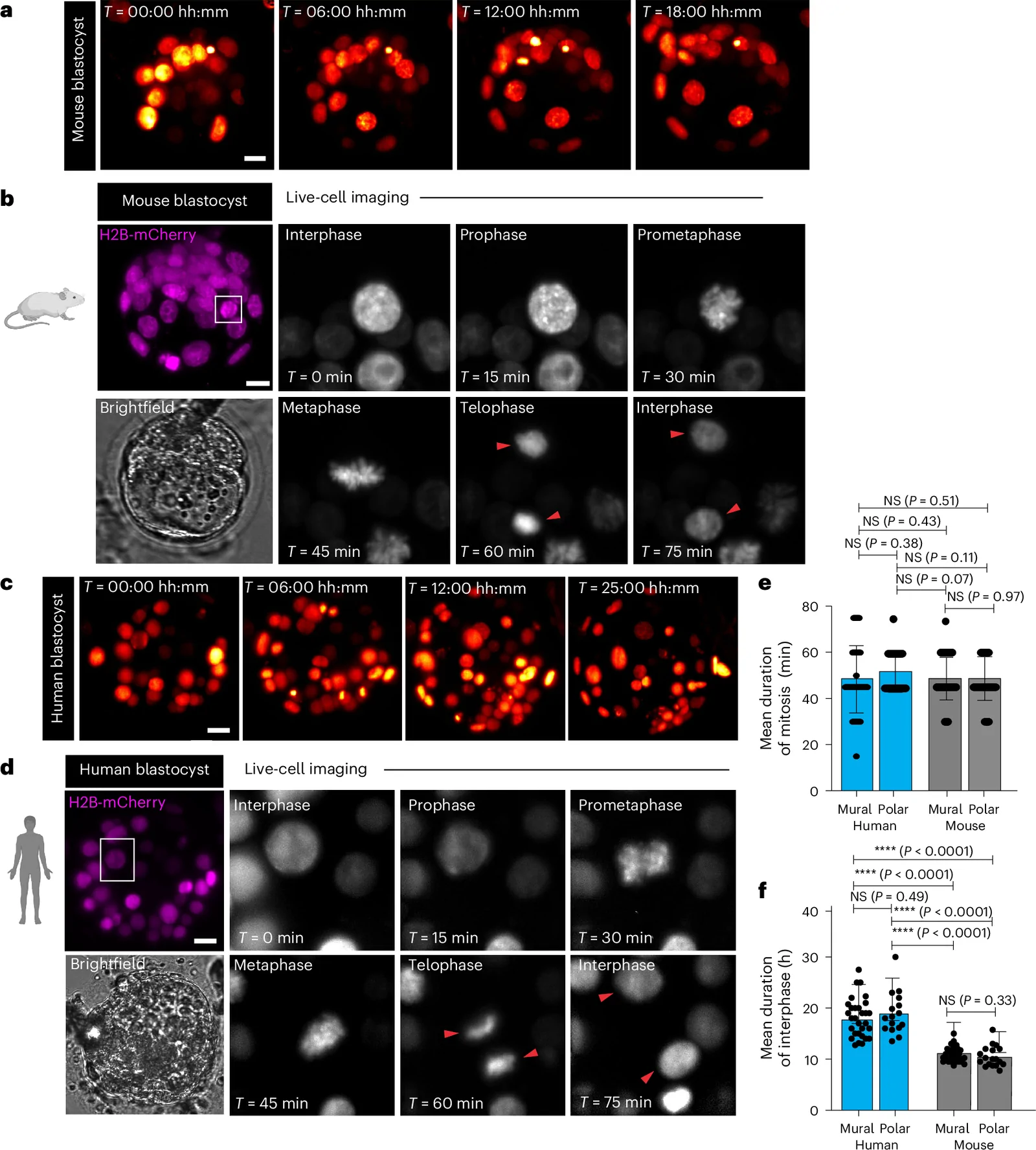
Live-embryo time-lapse imaging of mouse and human preimplantation embryos reveals differences in interphase duration. **a**, Live-embryo time-lapse light-sheet imaging of embryos labeled with H2B-mCherry enables the visualization of nuclei in mouse development. **b**, Time-lapse images of mitosis (prophase, metaphase, anaphase and telophase) in mouse cells at selected time points. **c**, Live-embryo time-lapse light-sheet imaging of embryos labeled with H2B-mCherry enables the visualization of nuclei in human embryo development. **d**, Time-lapse images of mitosis (prophase, metaphase, anaphase and telophase) in human cells at selected time points. Red arrowheads in **b** and **d** indicate the daughter cells resulting from mitosis. **e**, Quantification of the duration of mitosis in polar versus mural trophectoderm cells from mouse and human blastocyst-stage embryos (*n* *=* 17 mouse and *n* *=* 13 human blastocysts; *n* *=* 90 mitotic cells in mouse blastocysts and *n* = 90 mitotic cells in human blastocysts). **f**, Quantification of the duration of interphase in polar versus mural trophectoderm cells from mouse and human blastocyst-stage embryos (*n* *=* 17 mouse and *n* *=* 13 human blastocysts; *n* *=* 45 interphase cells in mouse blastocysts and *n* = 44 interphase cells in human blastocysts). Statistical analysis by two-tailed *t*-test. ****P < 0.0001. Error bars represent the mean ± s.d. Scale bar, 20 µm. See Supplementary Videos 1 and 2. *T*, time. Illustrations in **b** and **d** created using BioRender.com. Source data

Light-sheet imaging following nuclear DNA labeling with *H2B-mCherry* allowed us to track the phases and duration of mitosis (prophase, metaphase, anaphase and telophase) in mouse embryos (Fig. 2a,b and Supplementary Video 1). We subsequently thawed and electroporated early human blastocysts cryopreserved at 5 d postfertilization (dpf) with equivalent concentrations of *H2B-mCherry* mRNA and live imaged the embryos by light-sheet microscopy for up to 46 h (Fig. 2c,d and Supplementary Video 2). Mitosis was defined as the interval between a prophase and the first signs of telophase, and here we focused on comparing timing of mitosis in mouse and human blastocyst-stage embryos. We determined that there was a similar duration from the start to end of mitosis between the species, regardless of whether the trophectoderm cells were positioned overlaying the inner cells (polar) or surrounding the fluid-filled blastocoel cavity (mural). In human embryos, mural cells had a mean mitotic duration of 51.09 ± 11.11 min, while polar cells had a mean of 52.64 ± 9.13 min (*n* = 90 cells from 13 human embryos). In mouse embryos, mural cells exhibited a mean mitotic duration of 49.95 ± 8.68 min, and polar cells had a mean of 49.90 ± 8.32 min (*n* = 90 cells from 10 mouse embryos) (Fig. 2e). By contrast, we found that interphase was longer in mural and polar cells of human embryos compared with mouse embryos. The mean interphase duration in mural and polar human cells was 18.10 ± 3.82 h and 18.96 ± 4.15 h, respectively, whereas in mouse embryos, it was significantly shorter, with mean durations of 11.33 ± 3.14 h and 10.51 ± 2.03 h (Fig. 2f). This suggests that a difference in the timing of interphase is a contributing factor for setting the pace of preimplantation development across different species^23^.

### De novo chromosome segregation errors in in vitro cultured mouse and human blastocyst-stage embryos

**Fig. 3:**
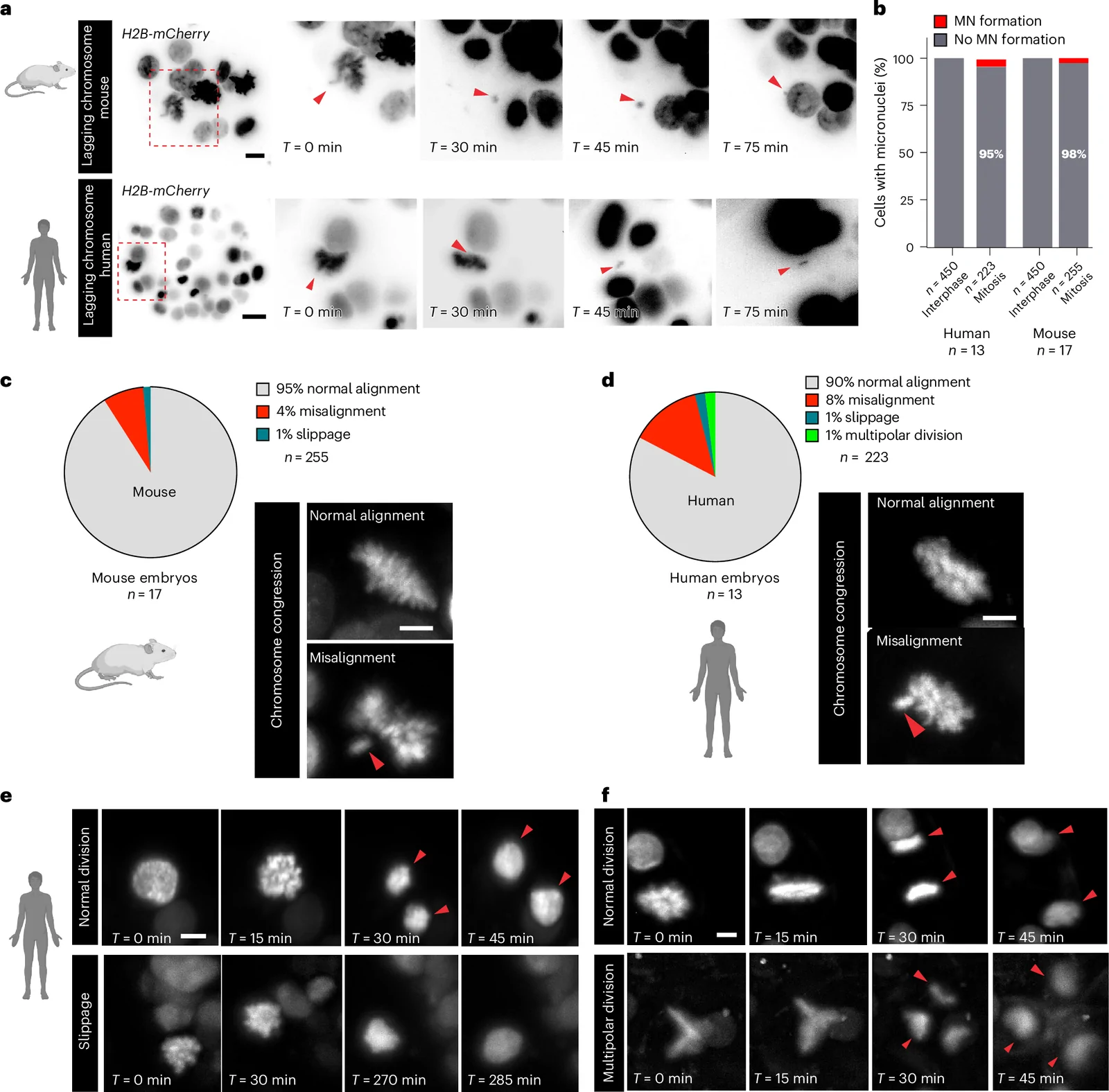
Chromosome segregation errors in mouse and human embryos at the blastocyst stage. **a**, Examples of live-embryo time-lapse light-sheet imaging of mouse and human embryos expressing *H2B-mCherry* mRNA, enabling the identification of misaligned chromosomes, lagging chromosomes and the formation of micronuclei. **b**, Quantification of micronuclei formation in mitotic and interphase cells. The percentage of cells with micronuclei was measured in both mitotic and interphase stages in mouse and human embryos. Micronuclei were assessed in 450 interphase cells and 223 dividing cells from 13 human embryos, and in 450 interphase cells and 255 dividing cells from 17 mouse embryos. **c**, Analysis of chromosome alignment before anaphase in H2B-mCherry–expressing mouse embryos (*n* *=* 17 mouse embryos; *n* *=* 255 mitotic cells). **d**, Analysis of chromosome alignment before anaphase in H2B-mCherry–expressing human embryos (*n* *=* 13 human embryos; *n* *=* 223 mitotic cells). **e**,**f**, Time-lapse imaging examples of mitotic slippage (**e**) and multipolar division (**f**) in human embryos compared with normal division at the selected times shown. Daughter cells are indicated with arrowheads. Scale bar, 20 µm. See Supplementary Videos 3–5. MN, micronuclei. Source data

**Extended Data Fig. 3:**
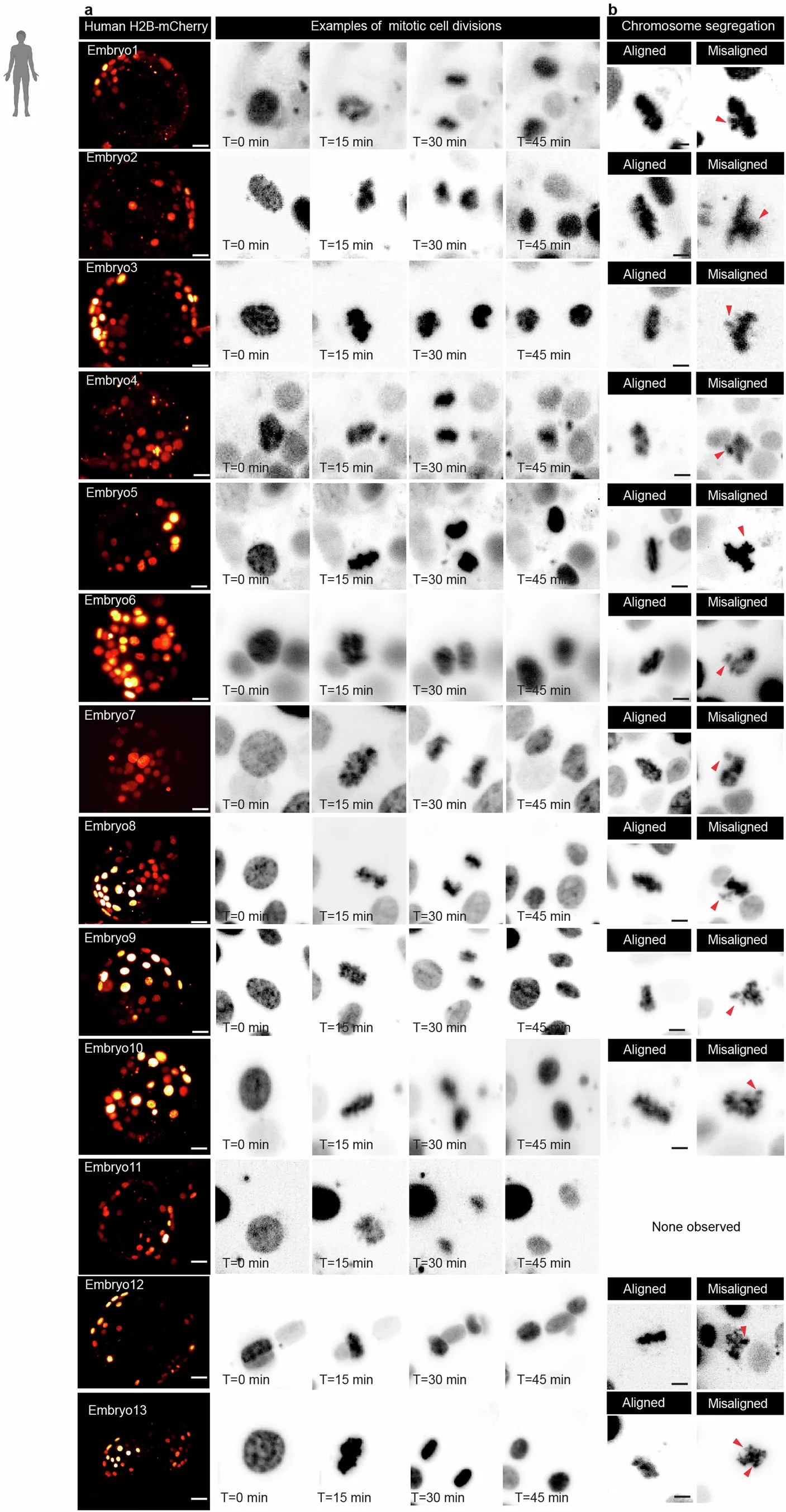
Light-sheet live imaging to visualise mitotic cell divisions in human embryos. **a**, Time-lapse images of mitosis (prophase, metaphase, anaphase and telophase) in human cells at the blastocyst stage. Human embryos were imaged following H2B-mCherry nuclear labelling (n = 13). **b**, Examples of normal alignment and misalignment of chromosomes observed in human blastocyst cells. Misalignment indicated with an arrowhead. Data from three independent experiments. Scale bar, 30 μm.

**Extended Data Fig. 4:**
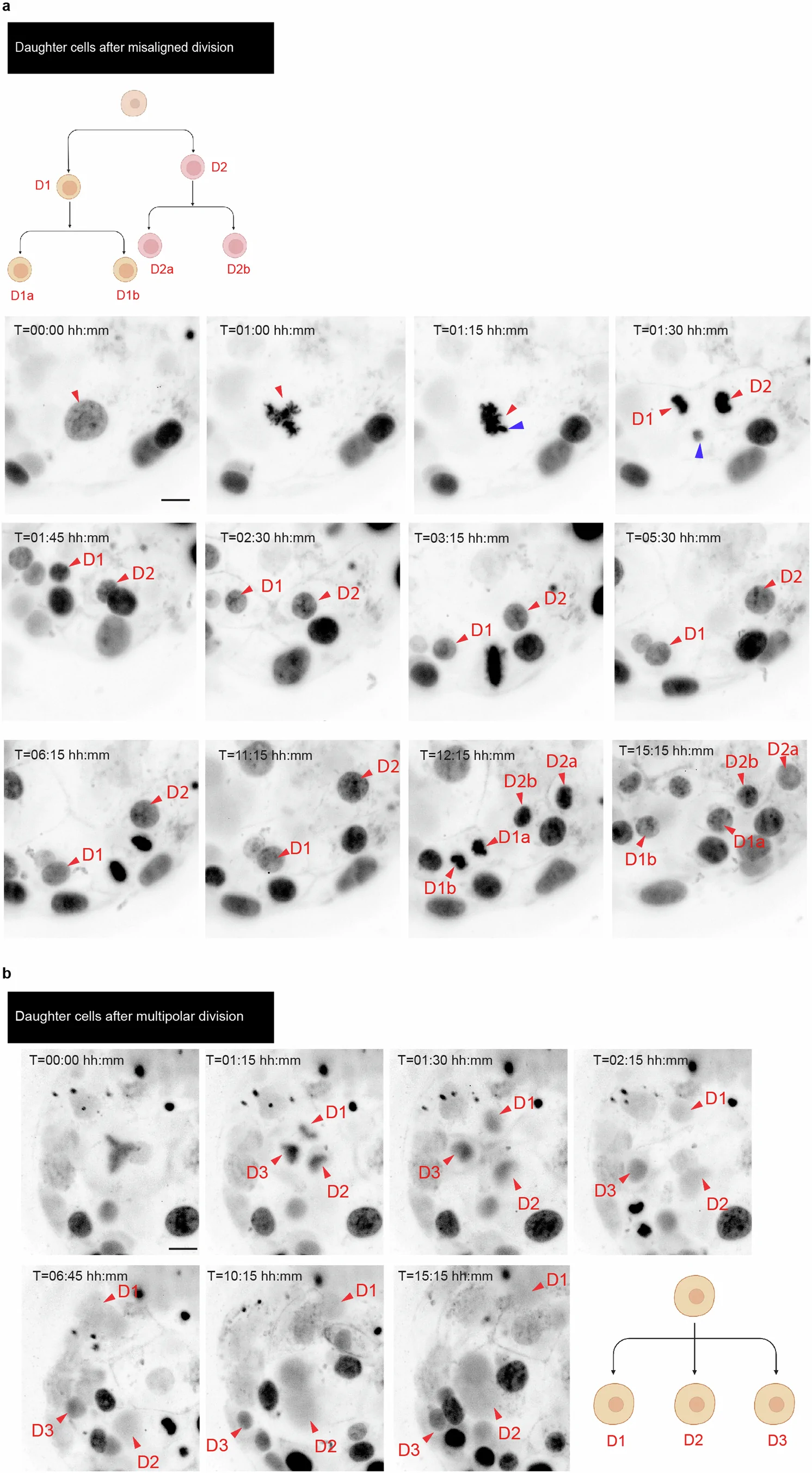
Fate of daughter cells following chromosome misalignment and multipolar division. **a**, Time-lapse images showing the fate of daughter cells after a misaligned chromosome division. Cells were tracked for 15 h to assess their viability and potential developmental outcome. **b**, Representative example of daughter cells following a multipolar division. Data from three independent experiments. Scale bar: 30 μm. Schematics in **a** and **b** created using BioRender.com.

We reasoned that our ability to label chromosomes and live image cell divisions would allow us to characterize chromosomal segregation errors and their consequences in blastocyst-stage human embryos. To identify mitotic errors, we analyzed the dynamics of chromosome segregation from 5 to 7 dpf in blastocyst-stage human embryos (*n* = 223 cell divisions across 13 labeled human blastocysts) and compared this with chromosome segregation in mouse embryos at equivalent stages from 3.25 to 4 dpf (*n* = 255 cell divisions across 17 labeled mouse blastocysts) (Fig. 3a–d). Before the onset of anaphase, chromosomes are fully aligned in 95% of mouse blastocyst-stage cells, while 90% of chromosomes in human blastocyst-stage cells are similarly aligned. Misaligned chromosomes were observed in 8% of human cells compared with 4% in mouse cells (Fig. 3c,d and Extended Data Figs. 3 and 4). This included lagging chromosomes and micronuclei formation in one of the daughter cells of human or mouse blastocyst-stage embryos. Micronuclei formation was observed exclusively during mitosis, with no detection of nuclear shedding during interphase, indicating that chromosomal abnormalities arise specifically during cell division rather than through nuclear fragmentation in interphase (Fig. 3a,b and Supplementary Video 3), consistent with previous studies of the first mitotic division in human embryos^19^.

We observed a rare event in 2 of 223 observed cell divisions in human blastocysts, whereby a trophectoderm cell prematurely exits from mitosis and, in the absence of chromosome segregation and cell division, enters the next G1 phase of the cell cycle as a tetraploid cell (Fig. 3e and Supplementary Video 4). This is a process referred to as mitotic slippage^24,25^. In mouse embryos, we observed slippage in 3 of 255 dividing trophectoderm cells, which we presume leads to tetraploidy. Notably, we observed de novo multipolar cell divisions in 2 of 223 dividing blastocyst-stage cells in human embryos. Multipolar spindle formation in anaphase resulted in the production of three daughter cells (Fig. 3f, Extended Data Fig. 4 and Supplementary Video 5). Although we cannot rule out that the three nuclei are within one or two cells without three-dimensional (3D) membrane segmentation, their migration suggests that they segregate to distinct cells. To investigate the consequences of these errors on cell viability, we tracked the fate of daughter cells arising from misaligned chromosomes. We found that these cells remained viable, undergoing at least one additional round of cell division (Extended Data Fig. 4a). Similarly, daughter cells from a multipolar division also remained viable throughout the remaining 15-h imaging period, suggesting that, despite severe mitotic errors, these cells retained their ability to persist and avoid death at this stage (Extended Data Fig. 4b). It remains unclear whether the mitotic slippage observed in blastocyst-stage human cells mirrors somatic cell mechanisms that bypass the spindle assembly checkpoint, leading to mitotic delay, cell death, aneuploid progeny or mitosis escape into G1^25,26^. Our findings suggest that misaligned chromosomes, multipolar division and slippage that arises de novo at the blastocyst stage in human embryos contribute to mosaic aneuploidy.

### Passive inheritance and reincorporation of micronuclei in mouse and human embryos

**Fig. 4:**
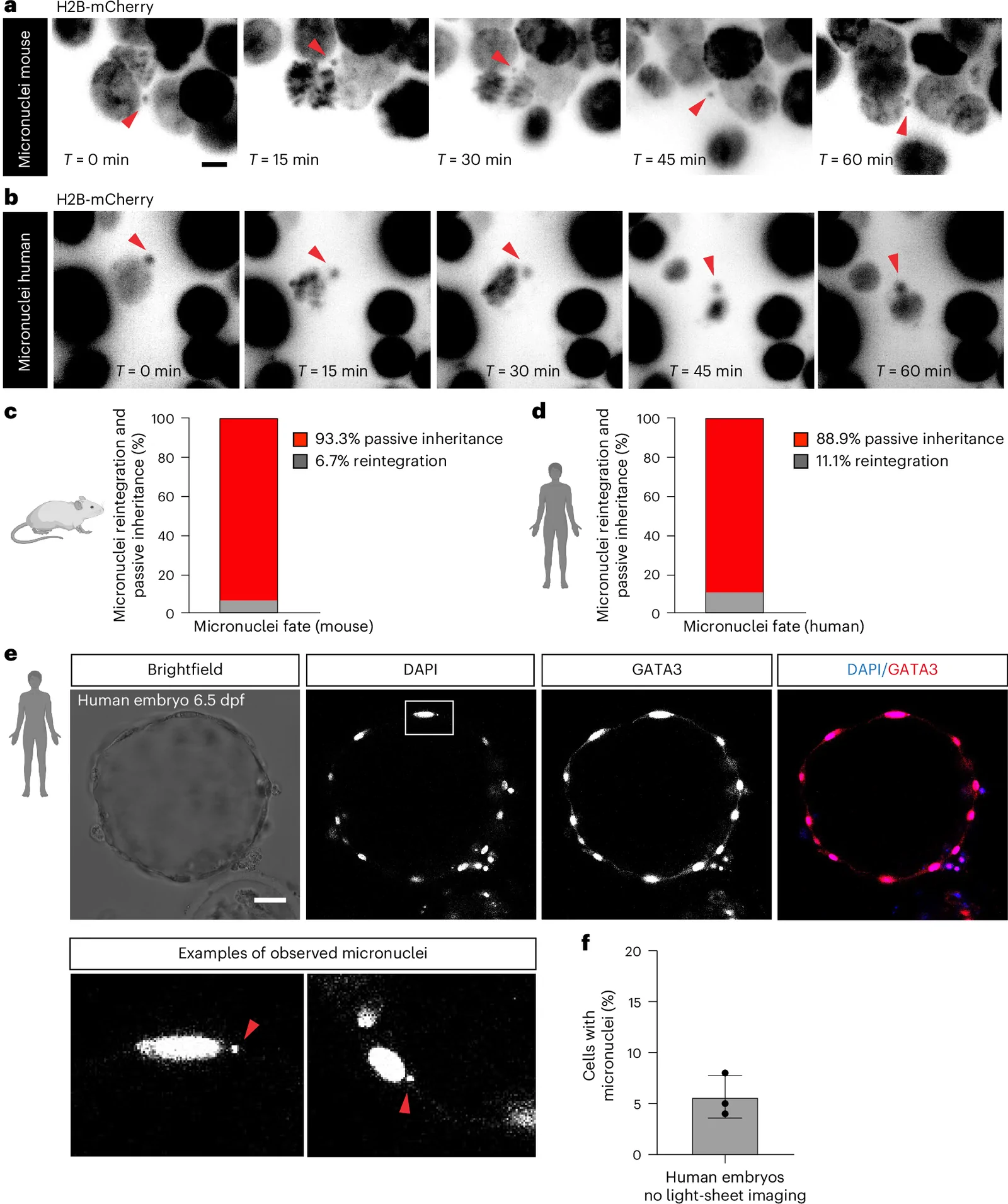
Micronuclei inheritance in mouse and human blastocysts. **a**,**b**, Example of micronuclei inheritance observed in H2B-mCherry-expressing mouse embryos (**a**) and in H2B-mCherry-expressing human embryos (**b**) during live-embryo time-lapse light-sheet imaging. Micronucleus (red arrow) stays distinct from the rest of the chromosomes throughout mitosis and was inherited by one of the daughter cells in mouse embryos and human embryos. Inverted images in black and white are used for better visualization. **c**, Percentage of micronuclei reintegration and passive inheritance in mouse embryos. Fifteen micronuclei were observed across 11 mouse embryos. **d**, Percentage of micronuclei reintegration and passive inheritance in human embryos. Nine micronuclei were observed in eight different human embryos. **e**, Human embryo cultured in a conventional incubator and not subject to light-sheet imaging indicating presence of micronuclei. Immunofluorescence analysis of a fixed human blastocyst at 6.5 dpf stained for GATA3 (trophectoderm molecular marker, magenta) and DAPI (blue) nuclear labeling. Data from two independent experiments, *n* = 3 total human embryos. **f**, Percentage of cells with micronuclei in human blastocysts cultured in a conventional incubator in the absence of light-sheet imaging. Micronuclei indicated with an arrowhead. Error bars represent the mean ± s.d. Scale bar, 30 µm. See Supplementary Video 6. Source data

**Extended Data Fig. 5:**
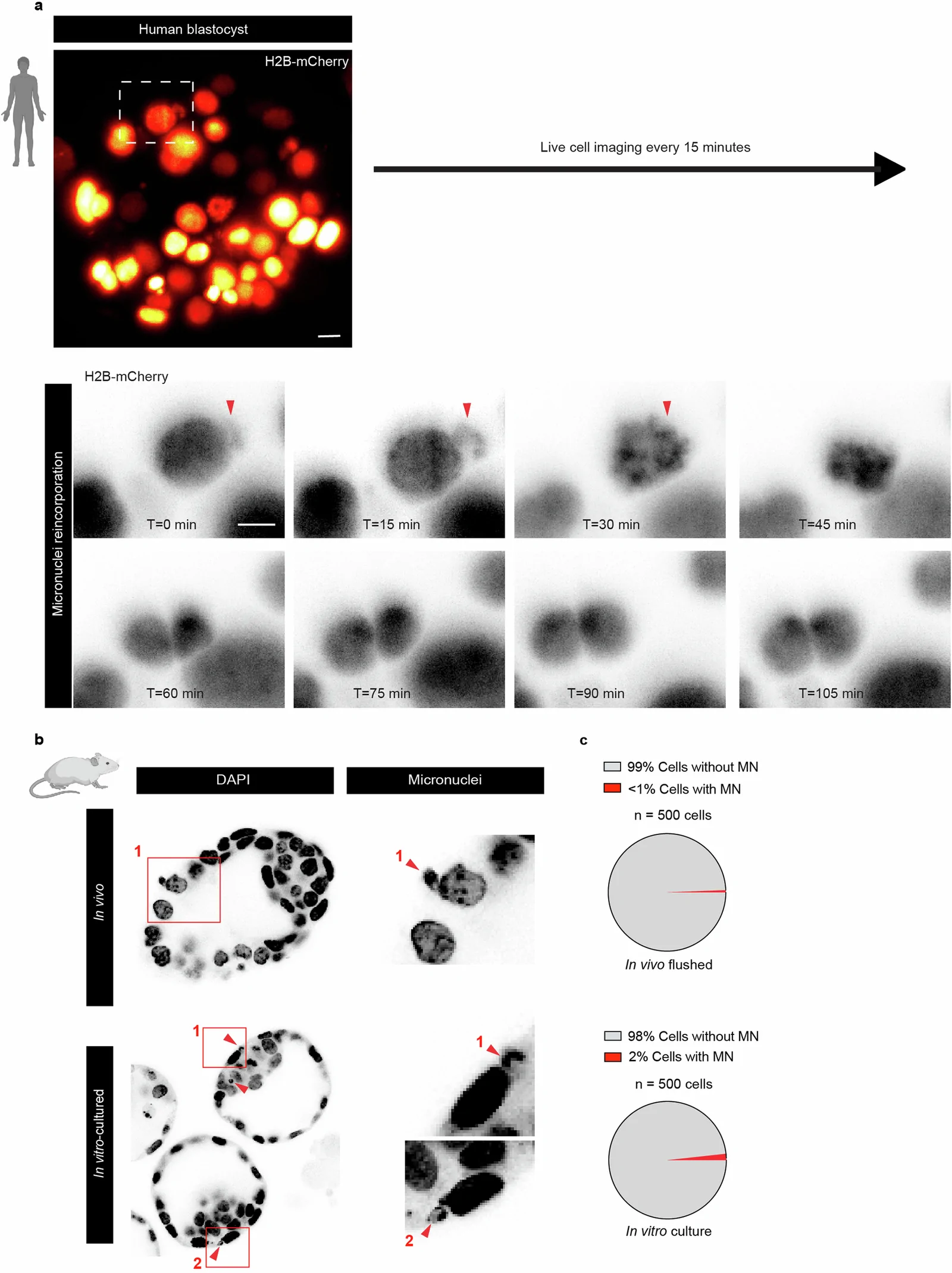
Micronuclei reincorporation during mitosis and their presence in in vitro cultured and in vivo flushed mouse blastocysts at day 4 post fertilization. **a**, Example of micronuclei reincorporating during mitosis in a human embryo expressing H2B-mCherry. Micronuclei indicated with an arrowhead. Scale bars, 15 μm. Data from three independent experiments **b**, Representative images of *in vitro*-cultured and *in vivo* flushed mouse blastocysts at embryonic day 4 stained for nuclear (DNA, blue) markers. Micronuclei were identified as small, extranuclear DNA structures distinct from the main nucleus (arrowheads). **c**, Quantification of micronuclei formation in blastocyst-stage embryos. A total of 500 cells from multiple embryos were analyzed. Data are presented as a pie chart, showing the percentage of cells containing micronuclei relative to the total number of cells analyzed. Data from two independent experiments. Scale bar, 30 μm. Source data

**Extended Data Fig. 6:**
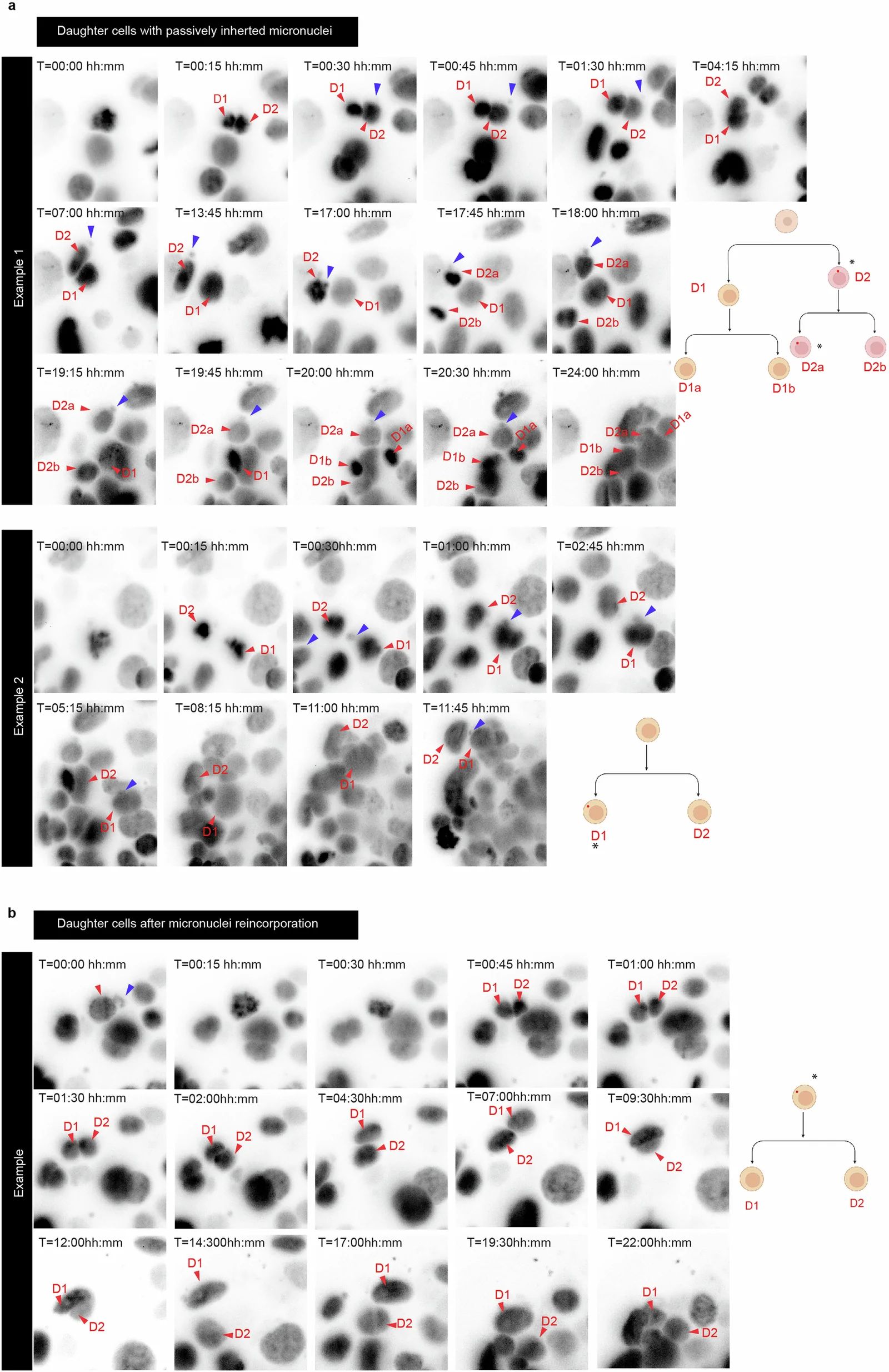
Fate of daughter cells following micronuclei formation. **a**, Representative time-lapse images showing two examples of daughter cells with passively inherited micronuclei (*). Example 1 was tracked for 24 h, while Example 2 was monitored for 12 h. **b**, Example of daughter cells in which micronuclei were reincorporated into the primary nucleus. Red arrowheads indicate daughter cells, and blue arrowheads indicate micronuclei. Data from three independent experiments. Scale bar: 30 μm. Schematics in **a** and **b** created using BioRender.com.

Micronuclei are frequently observed in cancer cells and in mammalian embryos, including humans and mice^27–30^. While the predominant fate of micronuclei is persistence in the cytoplasm and passive inheritance during subsequent mitosis in mouse embryos^28^, in cancer cells, chromosomes within micronuclei reintegrate into the nucleus. To understand the fate of micronuclei, we tracked what occurs after their formation in human and mouse blastocysts using live-embryo light-sheet imaging. Notably, the majority of micronuclei (89% in human and 93% in mouse) were maintained in the cytoplasm and did not appear to fuse with the nucleus during interphase (Fig. 4a–d). Following nuclear envelope breakdown, micronuclei remained separated from the chromosomes of the nucleus throughout the M-phase (Fig. 4a,b). The majority of micronuclei were consistently passively inherited in one of the daughter cells in both mouse and human embryos (Fig. 4c,d). Additionally, we also observed that micronuclei reintegrated in 11% of the cases in human blastocysts and in 7% of the cases in mouse blastocysts, suggesting a potential mechanism for genomic instability correction during early development (Extended Data Fig. 5 and Supplementary Video 6). Moreover, immunofluorescence analysis of human embryos cultured in conventional culture conditions, as well as flushed mouse blastocysts, shows that micronuclei were also detected in the absence of live-embryo light-sheet imaging (Fig. 4e,f and Extended Data Fig. 5b,c). By monitoring the fate of cells with micronuclei, we observed that the cells not only remained viable but also continued to proliferate, successfully producing daughter cells in the developing human blastocyst (Extended Data Fig. 6a,b). Altogether, these findings revealed a unique conserved pattern of micronuclei inheritance in mouse and human embryos.

### Technique for semi-automated segmentation and tracking shows that human trophectoderm cells are largely restricted at the blastocyst stage

The timing of extraembryonic trophectoderm, placental progenitor, specification occurs at the morula stage in 16-cell-stage mouse embryos using reporter labeling as well as live-embryo light-sheet microscopy^21,31^. Previous studies labeling human trophectoderm and either reaggregating only labeled cells, or aggregating labeled cells with unlabeled human embryos, suggested that human trophectoderm cells at 5 dpf are not yet irreversibly committed and can contribute to both trophectoderm and uncommitted inner cell mass cells that give rise to the embryonic epiblast and yolk sac progenitor cells^29^. However, disaggregation/reaggregation studies are not equivalent to lineage-tracing studies in unperturbed embryos because the disaggregation technique is known to impact on cell fate^32,33^.

**Extended Data Fig. 7:**
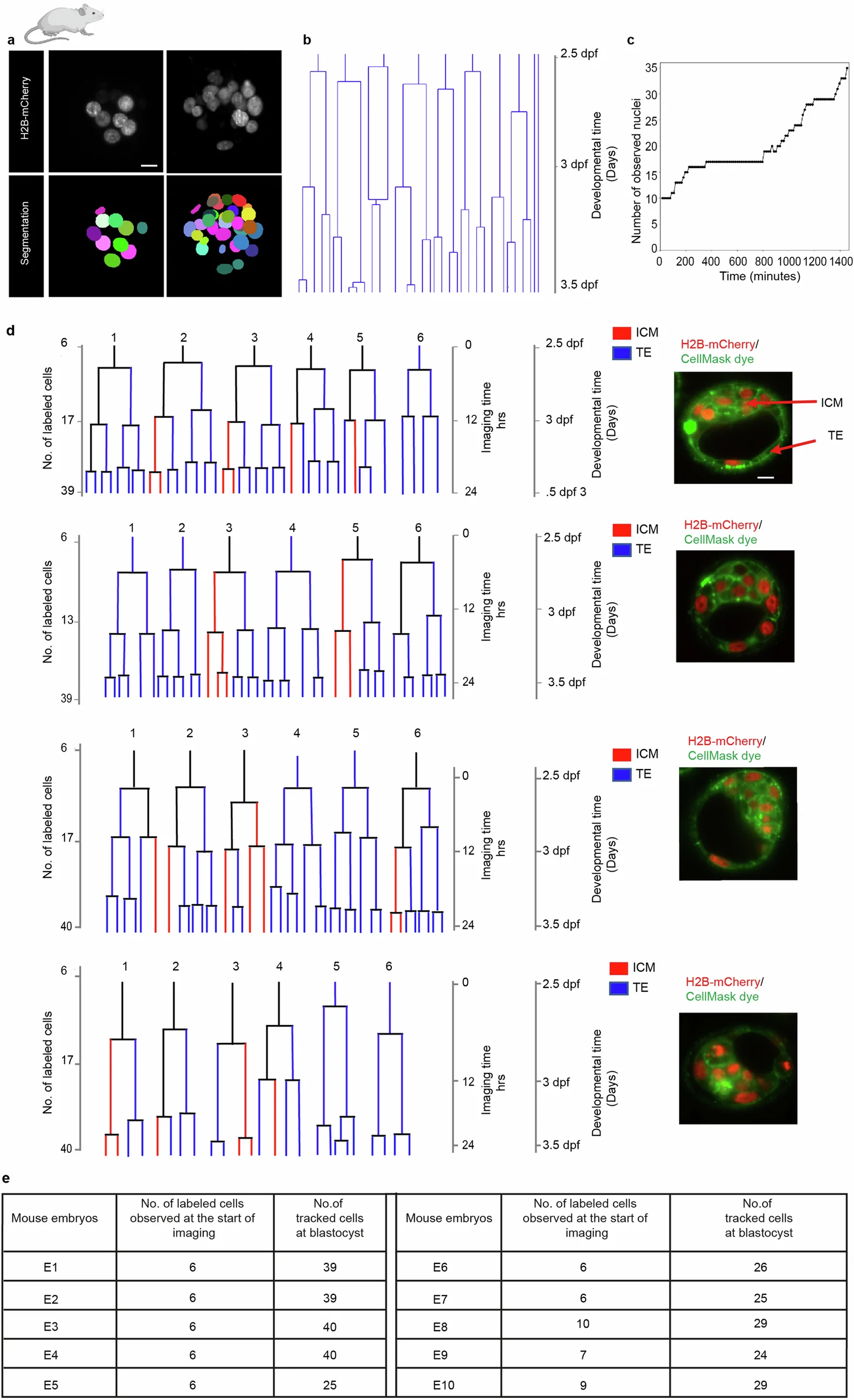
Lineage restriction in mouse preimplantation development. **a,b**, An example of 3D segmentation and tracking of mouse embryo nuclei following H2B-mCherry mRNA electroporation. **c**, Number of observed nuclei over time. **d**, Lineage trees of mouse embryos expressing H2B-mCherry from the 8-cell to blastocyst stage following light-sheet live embryo imaging. Inner cell mass (ICM, red) or trophectoderm (TE, blue) cells were assigned based on position and is displayed on the tree by color code. Representative z-section fluorescent image of the mouse embryos is shown for both H2B-mCherry and CellMask membrane dye. Scale bars, 15 μm. **e**, Table showing the number of labeled cells at the start of imaging and the number of cells successfully tracked over time. Labeled cells represent those expressing the fluorescent marker at the beginning of imaging, while tracked cells indicate the subset that could be reliably followed throughout the imaging period. Source data

To track and trace the eventual position of individual labeled nuclei in embryos, we initially developed a semi-automated nuclei segmentation method using a customized version of the StarDist-3D-based deep learning model with an optimized network for anisotropic 3D images (Extended Data Fig. 7a). To optimize input image quality, the dual-view light-sheet images were averaged for contrast enhancement, data resampled to match lateral and axial resolutions, intensity normalized and gamma corrected. Given the variability in the size and shape of nuclei, especially in human blastocysts, we altered the network architecture to increase the receptive field for segmentation. Training was performed on annotated mouse embryo images (Extended Data Fig. 7b,c). Nuclei in 3D time-lapse images were then automatically segmented, and tracking was performed using regularized Gaussian mixture optimal transport with added regularization to enhance the quality of automated tracking. The 3D segmentation and tracking were validated using Fiji’s TrackMate tool for manual correction. We initially trained the pipeline on annotated mouse embryo images (Extended Data Fig. 7b–e).

To determine whether outer trophectoderm cells are specified, we labeled mouse embryos at the cleavage stage (eight-cell stage at 2.5 dpf), then followed DNA-labeled nuclei in mouse embryos in early (3 dpf) and mid-blastocysts (3.5 dpf) and determined their final position within the blastocysts. Tracking mouse embryo cells showed that cleavage-stage blastomeres (2.5 dpf) give rise to daughter cells that contribute to both the trophectoderm and the cells of the inner cell mass (Extended Data Fig. 7d). By the early blastocyst stage (3 dpf) cells are specified to either trophectoderm or inner cell mass daughter cells (Extended Data Fig. 7d), consistent with previous studies^30,31,34–36^.

**Fig. 5:**
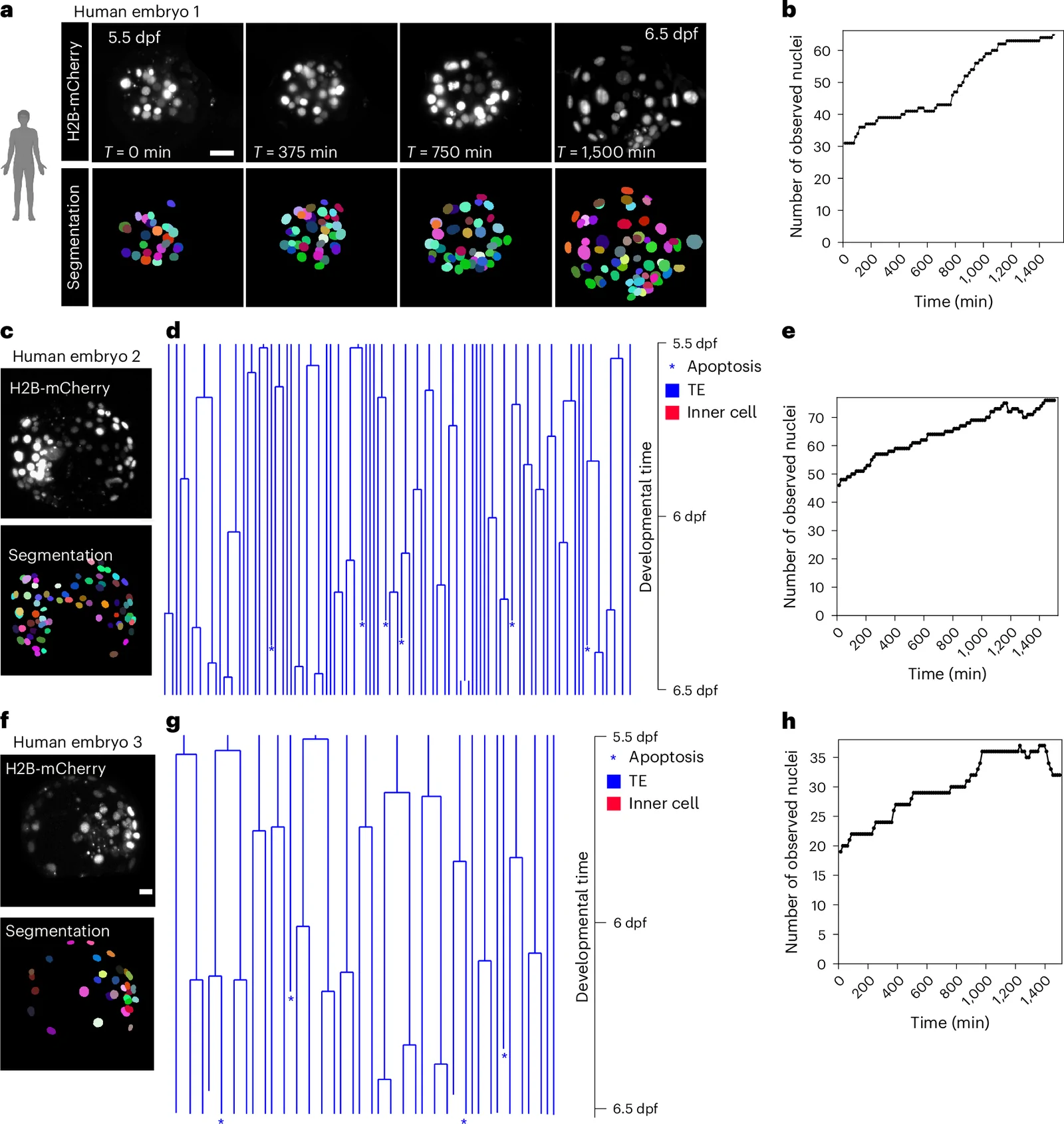
Trophectoderm cells are restricted in human blastocysts. **a**, Live-embryo time-lapse imaging and 3D segmentation of H2B-mCherry expression in human embryo 1 at different time points from 5.5 to 6.5 dpf at the selected time points indicated. The fluorescent signals of H2B-mCherry were used to track cells. **b**, Number of observed nuclei over the imaging period in human embryo 1. **c**, Selected frames from time-lapse imaging and 3D segmentation of H2B-mCherry expression in human embryo 2. **d**, Lineage tree of human trophectoderm-labeled cells at the blastocyst stage (human embryo 2). **e**, Number of observed nuclei over the imaging period in human embryo 2. **f**, Selected frames from time-lapse imaging and 3D segmentation of H2B-mCherry expression in human embryo 3. **g**, Lineage tree of human trophectoderm-labeled cells at the blastocyst stage (human embryo 3). **h**, Number of observed nuclei over the imaging period in human embryo 3. TE, trophectoderm. Scale bar, 30 µm. Source data

**Extended Data Fig. 8:**
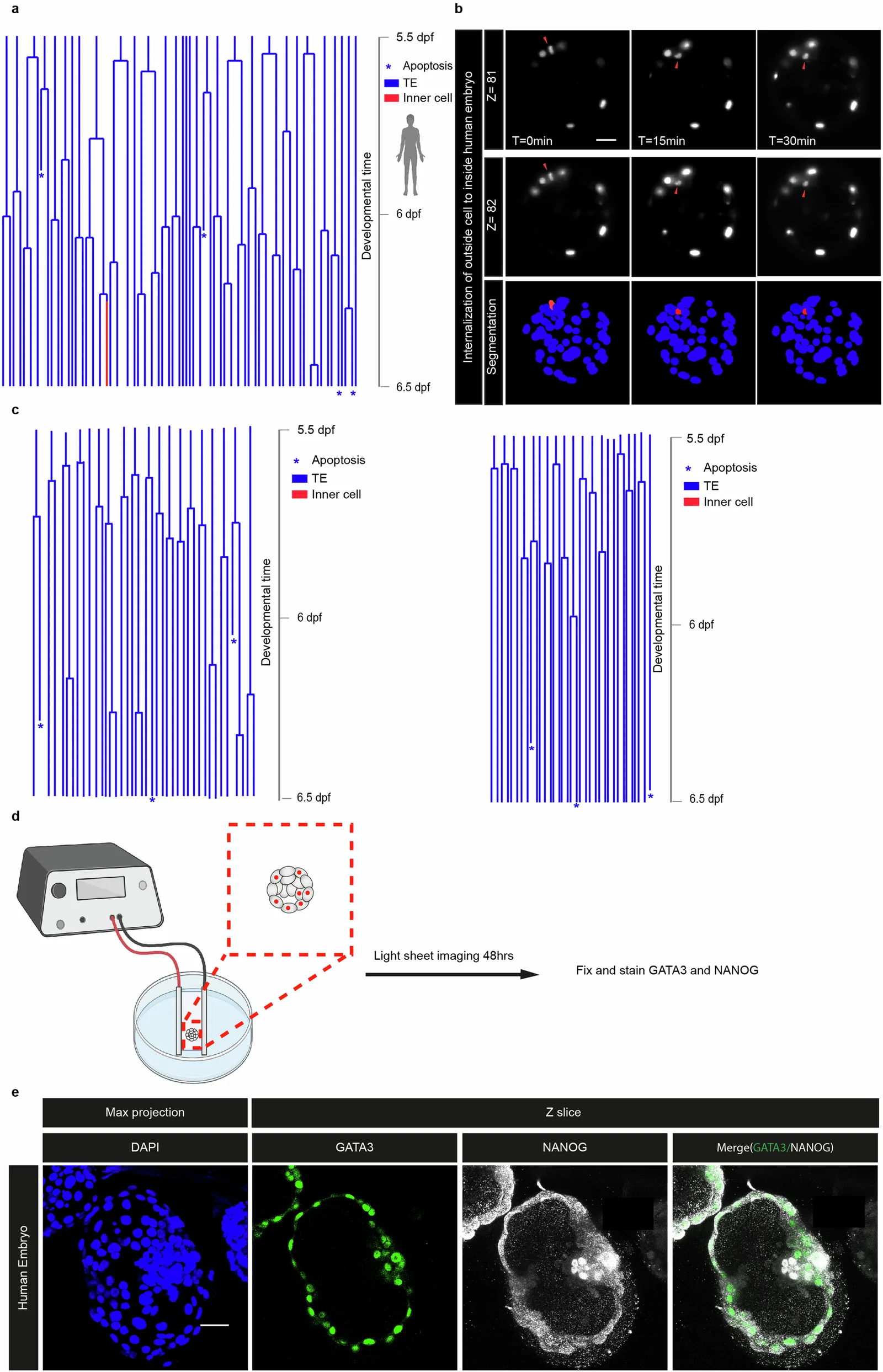
Evaluating trophectoderm lineage restriction in human embryos. **a**, Lineage tree of H2B-mCherry labelled nuclei of human pre-implantation blastocyst stage embryo. Each cell represented on the tree is color coded according to position. **b**, Selected frames from time-lapse imaging of an H2B-mCherry expressing human embryo. At the blastocyst stage, a transient-outer cell expressing H2B-mCherry was observed to migrate inward. The nucleus, marked with red arrow, signifies a transient outer cell moving inward. **c**, examples of lineage trees of human embryos expressing H2B-mCherry. **d, e**, human embryo electroporated with 500 ng/µl H2B mCherry mRNA were immunofluorescently analysed for the expression of NANOG (epiblast molecular marker), GATA3 (trophectoderm molecular marker) and DAPI nuclear staining. Data from two independent experiments. Scale bar: 30 μm. See Video S7, S8, S9, S10, S11. Illustrations in **d** created using BioRender.com.

We next tracked labeled cells in human embryos, which showed that the majority of outer trophectoderm cells remained on the outside and gave rise to more trophectoderm cells (Fig. 5a–h, Extended Data Fig. 8a–c and Supplementary Videos 7–9). Notably, in one embryo we observed a single cell at 6.25 dpf that was transiently positioned on the outside of the embryo, overlaying the inner cell mass, and once the cell divided, one daughter cell remained on the outside, while the other cell migrated to the inside position following cell division (Extended Data Fig. 8a,b and Supplementary Videos 7, 10 and 11). No further change in position was observed during imaging and it was assumed that the cell remained in the inside. After imaging, human embryos were fixed and stained for lineage-associated markers (Extended Data Fig. 8d,e). We detected the expression of molecular markers of the trophectoderm (GATA3) and epiblast (NANOG), indicating that light-sheet imaging did not obviously perturb human embryo development (Extended Data Fig. 8e). Moreover, we detected GATA3 expression in cells positioned inside the blastocoel cavity adjacent to the polar trophectoderm, consistent with a recent study^37^. Thus, we observed that it was rare for trophectoderm cells to change their position at the blastocyst stage of human development.

**Fig. 6:**
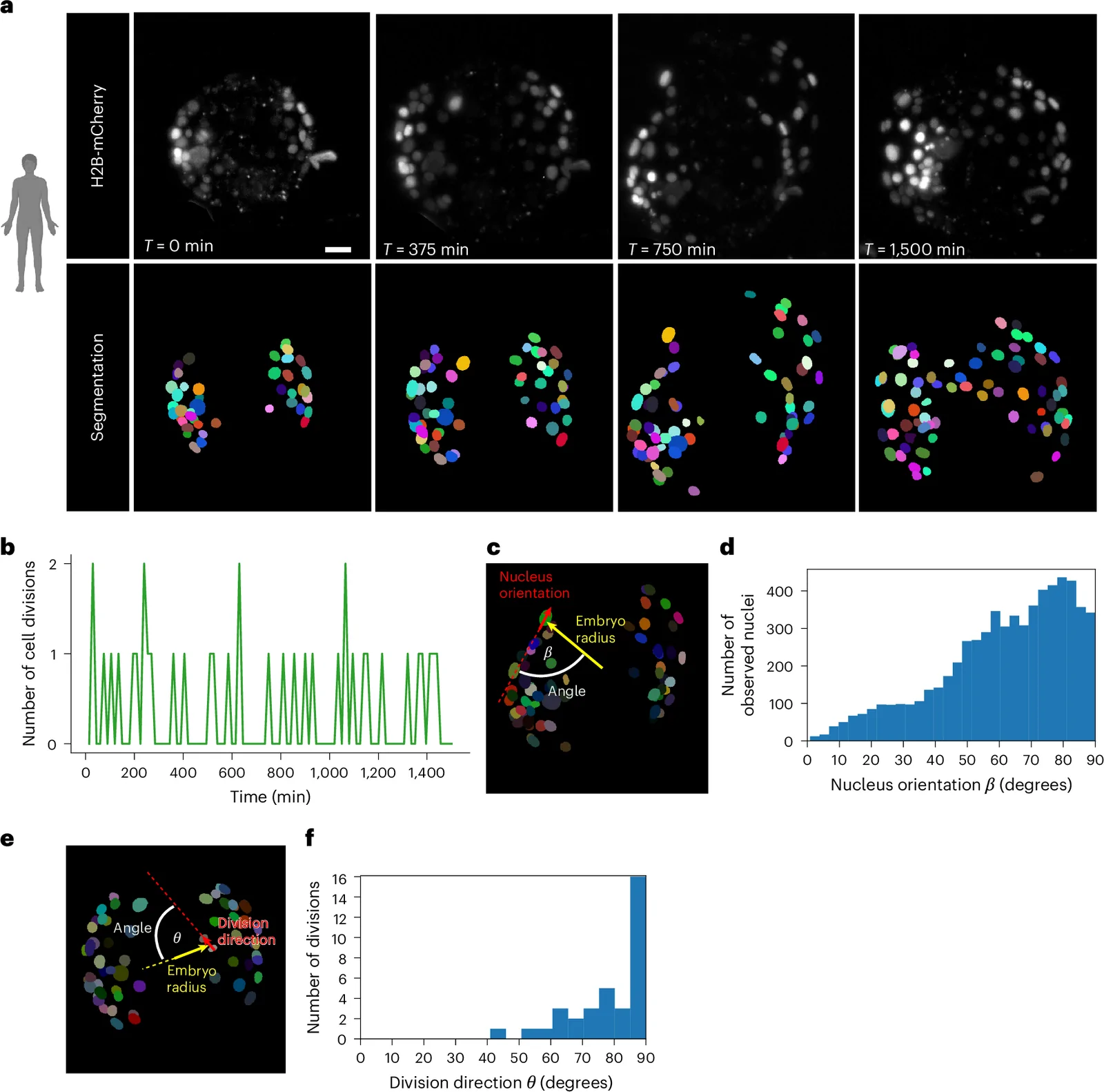
Nuclei shape, size and division axis orientation in human blastocysts. **a**, 3D segmentation of human embryo nuclei at different time points following imaging. **b**, Number of cell divisions across time. **c**, Schematic illustrating how the angle (*β*) of the nucleus orientation (red arrow) with respect to the embryo center (yellow arrow) was measured. **d**, Histogram of *β* across all time points showing that nuclei were oriented tangentially rather than radially to the center of the embryo (K-S test *P* < 10^−300^). **e**, Schematic illustrating how the angle (*θ*) between the embryo (yellow arrow) and nucleus (red arrow) orientation was measured. **f**, Histogram of *θ* across all time points showing that cells divide tangentially rather than radially to the center of the embryo (K-S test *P* ~ 10^−12^). Scale bar, 30 µm. Source data

**Extended Data Fig. 9:**
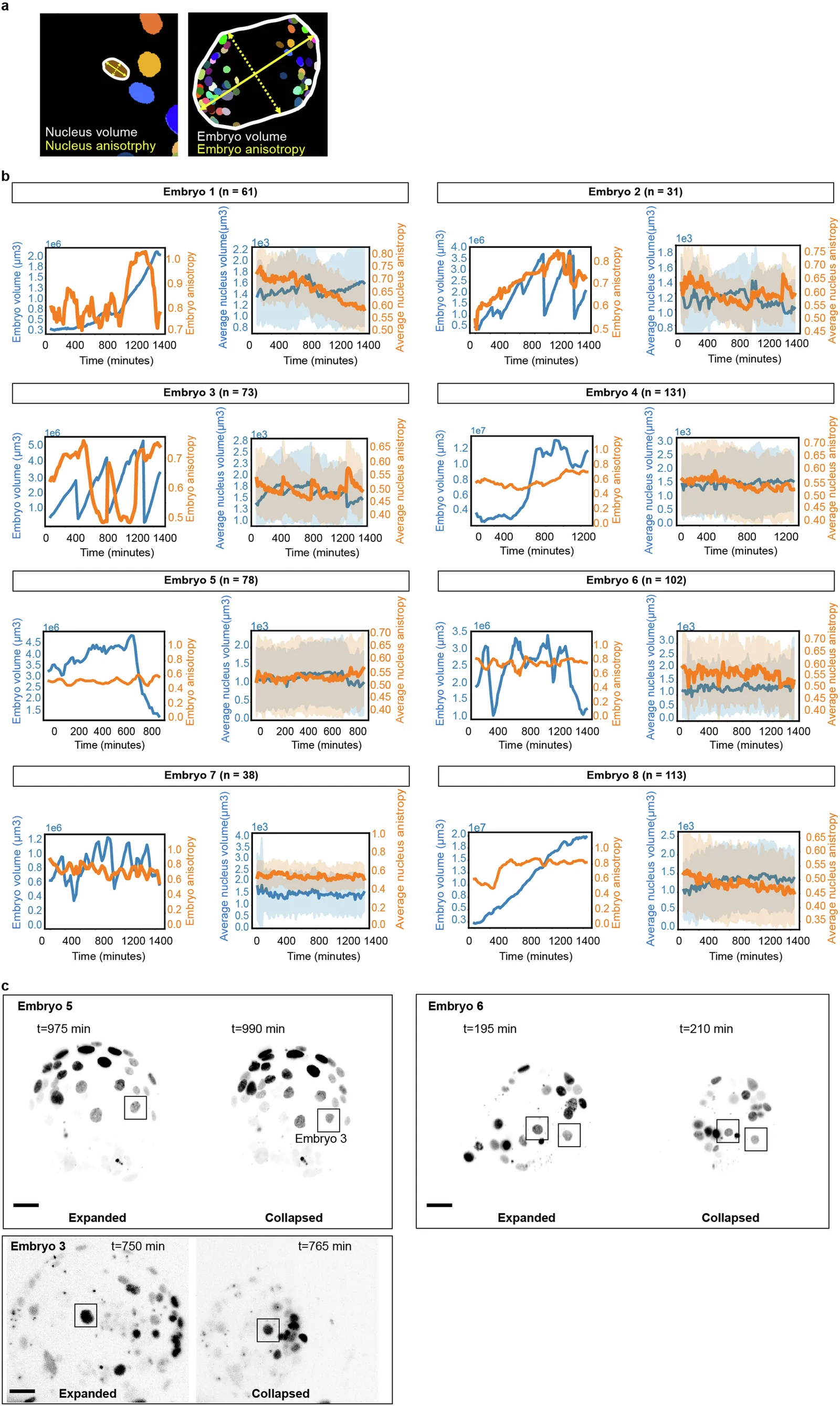
Nuclei shape, and size in human blastocysts. **a**, Schematic illustrating how embryo and nuclei size and shape were measured. **b**, Human embryo volume (blue) and anisotropy (orange), as well as average nuclear volume (blue) and anisotropy (orange), measured across developmental time from eight different embryos. “n” indicates the number of cells**. c**, Example images from different embryos highlighting changes in nuclear size in individual nuclei over time, particularly during periods of embryo collapse. Data from three independent experiments. Scale bar, 30 μm. Source data

Given that trophectoderm cells were largely restricted at the blastocyst stage, we next examined how changes in the size and shape of the human embryo impact on nuclear orientation and division which could further contribute to trophectoderm cell architecture and lineage restriction (Fig. 6a). We observed oscillations in the average volume and anisotropy of some of the human embryos over time as they underwent shape changes from a spherical to an elongated oblong blastocyst that hatched from a glycoprotein shell called the zona pellucida (Fig. 6a and Extended Data Fig. 9a,b). Notably, nuclear volume and anisotropy exhibited dynamic changes over time, such as when embryos underwent expansion or collapse (Extended Data Fig. 9b,c). We quantified nuclear orientation and division with respect to cell position within the embryo and found that the nuclei were oriented tangentially rather than radially (Kolmogorov–Smirnov (K-S) test *P* < 10^−300^) (Fig. 6c,d) in human embryos. Furthermore, an analysis of cell division angle also demonstrated a bias toward tangential division over radial division (K-S test *P* < 10^−9^) (Fig. 6e,f) in human embryos. These findings suggest that tissue geometry, through changes in nuclear orientation and division bias, may contribute to the maintenance of trophectoderm structure and lineage restriction.

## Discussion

Here we optimized a combination of methods for nuclear labeling and light-sheet microscopy to image human embryos up to 46 h of development with a focus on the blastocyst stage. This is considerably longer than the 12 h reported in previous publications using spinning disk microscopy^17,19^. Our live imaging of human and mouse embryos provides important insights into mitotic errors, micronuclei formation and inheritance as well as trophectoderm cell restriction at the blastocyst stage.

We identified species-specific differences in cell-cycle length between humans and mice. While the duration of mitosis remains relatively similar, interphase is longer in human embryo cells compared with mice. These findings are consistent with a previous study suggesting that the longer duration of human preimplantation development, compared with the mouse, is attributed to differences in cell-cycle length^17^. In the future it would be interesting to further investigate cell-cycle progression in both trophectoderm and inner cell mass using reporters of different stages of the cell cycle to have a more accurate timing of mitotic entry and exit^38^ and to understand which mechanisms explain the differences in interphase length between species.

In addition, our live imaging of human blastocysts revealed de novo mitotic chromosome segregation errors, including multipolar chromosome segregation and mitotic slippage, that may contribute to mosaic aneuploidy. Multipolar chromosome segregation may arise from supernumerary centrosomes, chromosomal instability or the loss of spindle pole integrity in cells with a normal number of centrosomes^34,35^. Occasionally, embryonic cells may acquire an extra diploid complement of chromosomes (leading to a tetraploid state) in human preimplantation embryos^36^. The precise origin of tetraploidy remains unclear, but potential explanations include cytokinesis failure and cell fusion^39^. Our data suggest that mitotic slippage also plays a role in driving tetraploidy in human embryos where cells experiencing a delay in mitosis exit without proper chromosome segregation to avoid cell death. We speculate that mitotic slippage may also provide an additional mechanism, besides endoreduplication, for the generation of tetraploid cells in mice^40–42^. Detailed analysis of centrosomes, spindle formation and the spindle checkpoint throughout human and mouse preimplantation development would inform the underlying causes of these mitotic errors.

Severe misalignment can result in lagging chromosomes which has been shown in previous studies to lead to micronuclei formation^27,28,43,44^. Using our live-embryo light-sheet imaging, tailored for prolonged imaging of mammalian preimplantation embryos, we traced the fate of micronuclei in both mouse and human embryos. Unlike cancer cells, our findings indicate that the majority of micronuclei are passively inherited by one of the embryonic daughter cells. This is consistent with previous studies investigating the mechanisms of micronuclei formation in mouse embryos^28^. Our findings also reveal that micronuclei in human blastocysts primarily arise during mitosis, rather than interphase, challenging a previous report that suggested nuclear shedding as a major source of micronuclei formation^17^.

Despite mitotic errors and micronuclei formation, human embryonic cells remain viable and continue dividing, which our data suggest contributes to mosaic aneuploidy at the blastocyst stage. This suggests that early embryonic cells possess distinct mechanisms for tolerating mitotic errors during preimplantation development, in contrast to somatic cells, where such errors typically trigger cell death^45–47^. Whether these cells are selectively eliminated or persist beyond preimplantation development remains an open question and would be important to determine. In somatic human cell lines and cancer cells, such chromosomes in micronuclei are subject to defective DNA replication and activate the interferon inflammatory signaling response through recognition by the viral receptor cyclic GMP-AMP synthase (cGAS)^48,49^. It remains unclear whether these events seen in somatic cells are similar in early mammalian development.

Our findings challenge the current practice of trophectoderm biopsies for preimplantation genetic testing for aneuploidy, a widely used clinical method to detect aneuploidy in human embryos^3,50^. It is generally assumed that the aneuploidies detected originate from earlier meiotic or mitotic errors. However, we provide evidence using advanced chromosome tracking and live-embryo imaging and segmentation technologies to show that de novo mitotic errors can arise late in human preimplantation development. Our data support the conclusion that these errors may be confined to the trophectoderm, leaving the inner embryo-fated cells unaffected. Our findings underscore the urgent need to reassess the clinical utility of preimplantation genetic testing for aneuploidy, as its widespread use may be limiting the transfer of viable embryos. Notably, our data strongly advocate for further research into the underlying cause and consequence of late-stage aneuploidies. Moreover, our findings suggest reconsideration of embryo transfer timing, because in vitro culture increases the risk of chromosome segregation errors.

Initiation of the trophectoderm transcriptional program, marked by GATA3 expression in outer cells, occurs at the morula stage and is conserved across species, including mouse, cow and human embryos^51,52^. By tracking cells expressing H2B-mCherry in human blastocysts, we observed the rare inward migration/internalization of an outer trophectoderm cell into the inner position, largely consistent with mouse blastocyst trophectoderm cells that remain committed to outside trophectoderm cells^30,31,53–55^. Unlike the trophectoderm of mouse blastocysts, trophectoderm cells of human, cow, pig and rabbit blastocysts have longer perdurance of molecular markers of the epiblast, including OCT4 and SOX2^56–58^. Human blastocyst aggregation assays using trophectoderm cells isolated from 5-dpf human embryos show that the trophectoderm cells are able to contribute to NANOG-expressing inner cell mass and can also give rise to blastocysts comprising trophectoderm and inner cell mass^29^, thereby suggesting plasticity at this stage. Additionally, in bovine embryos, morula aggregation assays suggest that trophectoderm cells retain the ability to give rise to the inner cell mass until at least the expanding blastocyst stage^56^. Altogether, this suggests that trophectoderm cells may not yet be irreversibly committed up to the blastocyst stage in human embryos, but further labeling and lineage-tracing studies are needed to determine the frequency of this, so far, rare observation.

Additionally, our findings from live imaging analysis are consistent with a recent study suggesting that trophectoderm cells in the late human blastocyst stage undergo multilayering and contribute to inner cells within the blastocoel cavity^59,60^. In the future, it will be important to characterize gene and protein expression of internalized cells to determine whether they express molecular markers of the trophectoderm, epiblast or yolk sac progenitor cells after live imaging using registration approaches, and to track and trace the cells for longer in development.

We also observed periodic cycles of blastocyst growth and collapse, which were accompanied by fluctuations in nuclear volume. These dynamic changes may be driven by mechanical forces, such as those arising from blastocyst expansion, hatching and collapse events. The current labeling method predominantly marks trophectoderm cells. As a result, it is not yet clear whether similar changes occur in cells of the inner cell mass at this stage. Simultaneous labeling of both DNA and the nuclear envelope in future studies will enable more precise monitoring of nuclear morphology and help distinguish between chromatin condensation and changes in nuclear size or shape. It will be interesting to determine the molecular mechanisms that regulate how human cells undergo cell fate determination and become irreversibly committed in their fate and function. The approaches we developed will be useful to address whether there is indeed bias of cells at the two-cell stage in human embryos, as has been recently suggested^61^. Altogether, we have developed methods for nuclear labeling, tracking and tracing of live human embryos, thereby revealing mechanisms of chromosome missegregation and early cell fate decisions. These methods can be used in the future in challenging-to-study and sensitive developmental contexts to investigate diverse cellular structures and to extend the time of live imaging of human embryos to gain further insights into early embryogenesis.

## Methods

### Ethics statement

This study was approved by the UK Human Fertilisation and Embryology Authority (HFEA), research license numbers R0162, R0397, R0401 and R0152, and independently reviewed by the Health Research Authority’s Research Ethics Committee projects 308099, 252286 and 272218.

The process of license approval entailed independent peer review along with consideration by the HFEA Licence and Executive Committees and the Research Ethics Committee. Our research is compliant with the HFEA Code of Practice and has undergone multiple inspections by the HFEA since the license was granted.

Informed consent was obtained from all couples that donated surplus embryos following infertility treatment. Before giving consent, donors were provided with information about the research project, an opportunity to receive counseling and the conditions that apply to the research license. The informed consent included approval of the publication of the results in scientific journals. No financial inducements were offered for donation. All donations were provided pseudonymized at the point of transfer to the research project. Embryos surplus to the patient’s treatment were donated cryopreserved and were transferred to the University of Cambridge and the Francis Crick Institute, where they were thawed and used in the research project. Further details about the research project that underwent ethical review can be found here: https://www.trophoblast.cam.ac.uk/Resources/embryo-donations

### Mouse embryo collection

Female mice, aged 4–8 weeks (C57BL6 × CBA), F1, were superovulated by intraperitoneal injection of 5 IU of pregnant mare serum gonadotrophin (Sigma-Aldrich), followed 48 h later with an intraperitoneal injection of 5 IU of human chorionic gonadotrophin (Sigma-Aldrich) and mating with 8-week or older (C57BL6 × CBA) F1 males. The mice were maintained under a 12-h light–dark cycle, ambient temperature 19/22 °C and humidity 45/65%. Zygotes were isolated from oviducts of plugged mice at 0.5 dpf in FHM medium (Merck; cat. no. MR-122-D) under mineral oil (Origio; cat. no. ART-4008-5P), and cumulus cells were removed using hyaluronidase (Sigma-Aldrich; cat. no. H4272). Alternatively, CD1 female and male mice were time mated and blastocysts were collected at 4 dpf by flushing uteri with FHM medium. Blastocysts were immediately fixed in 4% paraformaldehyde. All procedures involving animals were conducted in accordance with the UK Home Office, license number PP8826065.

### Viral transduction

Mouse embryos were transduced with Cellight Histone 2B-GFP (cat. no. C10594). Live-embryo imaging was conducted after 24 h using a confocal microscope. For AAV6 transduction, embryos were co-cultured with scAAV6-tdTomato (plasmid no. 59462) at varying concentrations: 1 × 10^8^ IU ml^−1^, 1 × 10^10^ IU ml^−1^, 1 × 10^12^ IU ml^−1^, or without AAV6, for 24 h. Subsequently, the embryos were cultured in vitro, and the expression of tdTomato was assessed after 24 h and 48 h using fluorescence microscopy. For lentivirus transduction, mouse embryos were transduced with a high-titer lentivirus carrying an H2B-GFP reporter (Addgene, plasmid no. 26777) and imaged after 24 h using a confocal microscope.

### Live-embryo staining

Mouse embryos were cultured in KSOM medium (Merk; cat. no. MR-101-D) supplemented with various dyes at working concentrations: SPY650-DNA (1:1,000; Spirochrome), Nuclight Rapid Red (1:1,000; Incucyte; cat. no. 4717), 1 μM 5-TMR-Hoechst, 1 μM 4-TMR-Hoechst, 1 μM 4-580CP-Hoechst and 1 μM 5-580CP-Hoechst^62^ (gift from the G. Lukinavičius lab) and were subsequently imaged after 48 h.

### Generation of modified mRNAs by in vitro transcription

mRNA synthesis was performed as previously described^57^. The double-stranded DNA template (H2B-mCherry plasmid no. 20972 from Addgene) was linearized, and a small aliquot of the digestion mix was subjected to gel electrophoresis to verify complete digestion. Linearized plasmid was purified using a PCR purification kit (Qiagen; cat. no. 28104). Poly(A) tailing was carried out using KAPA PCR ready mix (2×) and the following primer sets: the forward primer (CTTACTGGCTTATCGAAATTAATACGA) and the reverse primer (TTTTTTTTTTTTTTTTTTTTTTTTTTTTTTTTTTTTTTTTTTTTTTTTTTTTTTTTTTTTTTTTTTTTTTTTTTTTTTTTTTTTTTTTTTTTTTTTTTTTTTTTTTTTTTTTTTTTTTTTTTTTTTTTAAACAACAGATGGCTGGCAACTAGAAGG) from Integrated DNA Technologies. Subsequently, the digested plasmid was adjusted to a concentration of 10 ng μl^−1^. Tail PCR was run for 32 cycles and purified using a PCR purification kit. In vitro transcription was performed using MEGAscript T7 kit (Thermo Fisher; cat. no. AMB13345): a custom NTP mix was prepared with 3′-O-Me-m7G cap analog (60 mM; NEB), GTP (75 mM; MEGAscript T7 kit), ATP (75 mM; MEGAscript T7 kit), Me-CTP (100 mM; TriLink; cat. no. N-1014-1) and pseudo-UTP (100 mM; TriLink; cat. no. O-0263). The reaction was heated at 37 °C for 2 h. Then, 2 μl of Turbo DNase (Thermo Fisher; cat. no. AM2238) was added and incubated at 37 °C for 15 min. The DNAse-treated reaction mix was purified using RNAeasy kit (Qiagen; cat. no. 74104) according to the manufacturer’s instructions. RNA was phosphatase-treated using Antarctic phosphatase (New England BioLabs; cat. no. M0289S) and purified using MEGAclear kit (Thermo Fisher; cat. no. AM1909).

### Human and mouse embryo culture

**Extended Data Fig. 10:**
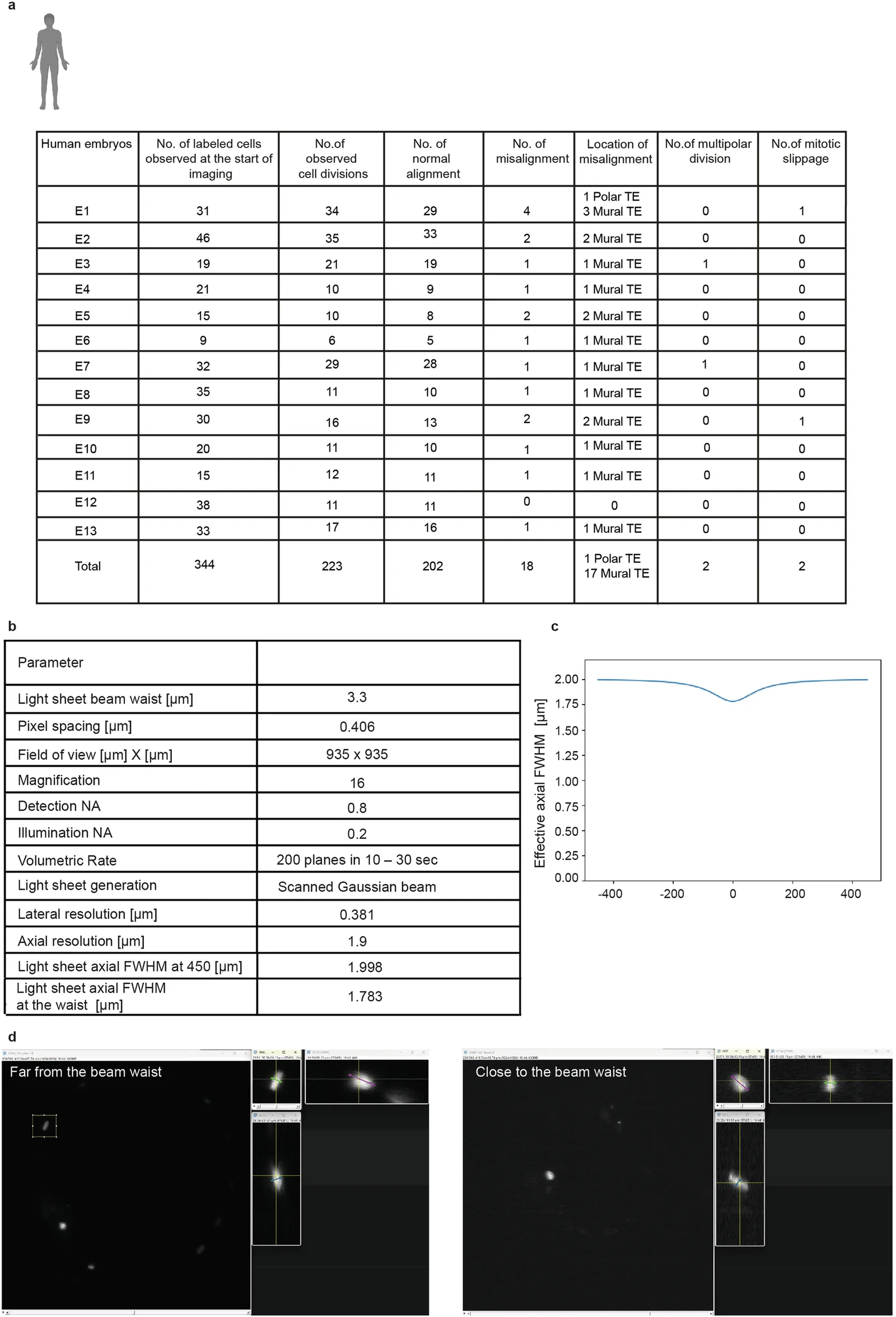
Number of labeled cells in human embryos and parameters of light-sheet imaging in our microscope. **a**, Table showing the number of labeled cells observed at the start of imaging, observed cell divisions, cells exhibiting normal alignment, and the number and location of cells exhibiting misalignment during the imaging. **b**, Table summarizing the microscope settings used for light-sheet imaging. **c**, Effective axial resolution along the light sheet. **d**, Example images of cells located near the beam waist and far from the beam waist, illustrating resolution.

Slow-frozen human embryos were thawed using the Blast thaw kit (Origio; cat. no. 10542010A), following the manufacturer’s instructions. Vitrified human embryos were thawed using Vit Kit-Thaw (Fujifilm; cat. no. 90137-SO). Mouse embryos and human embryos were cultured at 37 °C and 6% CO_2_ in drops of pre-equilibrated Global medium (LifeGlobal; cat. no. LGGG-20) supplemented with 5 mg ml^−1^ protein supplement (LifeGlobal; cat. no. LGPS-605) and covered with mineral oil (Origio; cat. no. ART-4008-5P). All human embryos used in the study are summarized in Extended Data Fig. 10a.

### Immunofluorescence and confocal imaging

Embryos were fixed using freshly prepared 4% paraformaldehyde in PBS either at room temperature for 1 h or overnight at 4 °C, followed by three washes in 1 × PBS with 0.1% Tween-20 (Sigma-Aldrich; cat. no. P1379-25ML) to eliminate residual paraformaldehyde. Subsequently, embryos were permeabilized with 1 × PBS with 0.5% Triton X-100 and then placed in blocking solution (3% BSA (Sigma-Aldrich; cat. no. 05470-5G) in 1 × PBS with 0.2% Triton X-100 (Sigma-Aldrich; cat. no. X100-5ML)) for 2 h at room temperature on a rotating shaker. Embryos were then incubated overnight at 4 °C on a rotating shaker with primary antibodies diluted in blocking solution, at the following concentrations: primary antibodies used were CDX2 (BioGenex; cat. no. MU392A-UC) at dilution 1:50, NANOG (2B Scientific; cat. no. REC-RCAB000lP) at dilution 1:100, NANOG (R&D; cat. no. AF1997) at dilution 1:200 and GATA3 (Abcam; cat. no. ab199428) at dilution 1:100. The following day, embryos were washed in 1 × PBS with 0.2% Triton X-100 for 20 min at room temperature on a rotating shaker and then incubated with 1:200 secondary antibodies diluted in blocking solution for 1 h at room temperature on a rotating shaker in the dark. Secondary antibodies were Donkey anti-Mouse Alexa Fluor 488, 594 or 647; Donkey anti-Rabbit Alexa Fluor 488, 594 or 647; and Donkey anti-Goat Alexa Fluor 488, 594 or 647 (Thermo Fisher). Next, embryos were washed in 1 × PBS with 0.2% Triton X-100 for 20 min at room temperature on a rotating shaker. Finally, embryos were placed in 1 × PBS with 0.1% Tween-20 with Vectashield and DAPI mounting medium (Vector Lab; cat. no. H-1200) (1:30 dilution). Embryos were placed on µ-Slide eight-well dishes (Ibidi; cat. no. 80826) for confocal imaging. Confocal immunofluorescence images were taken with an SP8 confocal microscope (Leica Microsystems) and 2-μm-thick optical sections were collected.

### Electroporation

Mouse and human embryos were washed and placed in drops of Opti-MEM (Thermo Fisher; cat. no. 31985062). The dish was then placed on a heated microscope stage (Olympus IX70). We transferred 7 µl of the H2B-mCherry mRNA solution onto an electroporation chamber between the electrodes of the plate (NEPA GENE, Sonidel; cat. no. CUY501P1-1.5). The impedance was adjusted to between 0.19 Ω and 0.21 Ω (typically 0.20 Ω) by either adding or removing the electroporation solution. The electroporation parameters of mouse embryos were as follows: 1 poring pulse of 0.1 V, lasting 50 ms with a 50-ms interval, 10% decay, immediately followed by 2 transfer pulses of 20 V, 40% decay, lasting 25 ms with a 50-ms interval. The electroporation parameters for human embryos were as follows: 6 poring pulses of 15 V, lasting 2 ms with a 50-ms interval, 10% decay, immediately followed by 5 transfer pulses of 5 V, 40% decay, lasting 50 ms with a 50-ms interval. Immediately after electroporation, the embryos were removed from the electroporation chamber, washed and cultured in equilibrated Global medium (LifeGlobal; cat. no. LGGG-20) supplemented with 5 mg ml^−1^ protein supplement (LifeGlobal; cat. no. GHSA125) and covered with mineral oil.

### Live-embryo imaging

Electroporated embryos were placed in a fluorinated ethylene propylene foil microwell sample holder, containing equilibrated Global medium (LifeGlobal; cat. no. LGGG-20), supplemented with 5 mg ml^−1^ protein supplement (LifeGlobal; cat. no. LGPS-605) and covered with mineral oil (Origio; cat. no. ART-4008-5P). We primarily used the LS2-Live dual illumination (Leica Microsystems) and inverted detection microscopes were used for live imaging embryos (in one human embryo, Embryo 1, we used the Viventis LS1 microscope which has single illumination). Light-sheet images were generated by two Nikon ×10, 0.2 numerical aperture (NA) illumination objectives. The illumination beam reaches the sample at an angle of 30° with the horizontal axis crossing air–glass and glass–water interfaces^63^. Beam waist of 3.3 µm was selected. Time-lapse images of embryos were captured every 15 min for up to 2 d at 37 °C and 6% CO_2_ with either a ×16, 0.8 NA or a ×25, 1.1 NA objective. A volume of 150–200 μm was acquired with a *Z* spacing of 2 μm between slices and 100-ms exposure time for each slice. Laser intensity was minimized to obtain a reasonable signal-to-noise ratio from the raw data while minimizing phototoxicity. Details of the microscope settings are provided in Extended Data Fig. 10b,c. The reconstruction of videos was performed using IMARIS software v.9.9 (Bitplane, AG) and Fiji ImageJ open-source image processing package.

### 3D nuclear segmentation and tracking

Semi-automated 3D nuclear segmentation and tracking was performed using (1) STAR-3D, a custom version (https://github.com/akarsa/anisotropic_stardist_3d) of the StarDist-3D network^58^ tailored to our data, and (2) Optimal3dTracks, a regularized Gaussian mixture optimal transport bases method^64^.

Both softwares are available for download (https://github.com/akarsa/star-3d, https://github.com/akarsa/optimal3dtracks).

As a first preprocessing step, averaging of dual-view light-sheet images was carried out, where available, for contrast enhancement. Furthermore, to avoid over-segmentation and account for variations in imaging parameters, we resampled the data to an optimized 0.381-µm lateral and 1.9-µm axial resolution, normalized the intensity and applied gamma correction. Samples suffering from more significant axial degradation were further downsampled axially. Model-based deconvolution^65^ was also used as an alternative. The StarDist-3D network architecture was altered to increase the receptive field of the network to cope with the increased size and shape variability in human embryo data. This was trained on annotated mouse embryo images (https://github.com/akarsa/star-3d).

Nuclei were automatically segmented over 3D time-lapses of 100 time points. Besides nuclei, the custom StarDist-3D network also delineates various unwanted, nuclei-like structures such as dirt in and around the embryo. As a pre-filtering step, features smaller than 30 µm^3^ in size were removed.

The segmented nuclei were tracked using regularized Gaussian mixture optimal transport (GMMOT)^64^ (https://github.com/akarsa/optimal3dtracks, https://github.com/judelo/gmmot) to calculate transition probabilities between positions at consecutive time points. A regularization term was added to GMMOT to improve the quality of the automated tracking.

Using an in-house Python script, the automatically generated 3D segmentations and tracks were converted to a format readable by Fiji’s TrackMate tool^66^. An expert biologist (A.A.) manually curated and corrected the results in TrackMate.

Videos of two-dimensional axial projections of both the intensity images and segmentations are provided in Supplementary Information; tracks were indicated by preservation of label color over time. The intensity images were masked to better visualize the region of interest. The videos also indicate the evolutions of the dendograms, and number of nuclei detected in the embryo. Several metrics were calculated to evaluate and correlate the sizes, shapes and orientations of the embryo and the segmented nuclei. For each segmented nucleus, the volume, anisotropy (computed as ratio between longest axis diameter and shortest axis diameter of the best fitting ellipsoid describing the nuclei), centroid and orientation (3D direction of the long axis) were calculated. The same statistics were computed for the entire embryo, that is, the convex hull of all segmented nuclei, at each time point. From these metrics, the ‘average shell thickness’ (standard deviation of distance between the nuclei and the embryo center), angle between the embryo and nuclei orientations (*α*), nuclei orientation with respect to the embryo center (*β*) and cell division direction (*ϑ*) with respect to the embryo center^21^ were also computed. Linear regression analysis was used to investigate correlations between different measures, and K-S test was performed to statistically evaluate the deviations of *β* and *ϑ* from the uniform distribution.

### Quantifications and statistical analysis

Statistical details for each experiment are described in the corresponding figure legends. Biological replicates and number of cells analyzed are denoted as ‘*n*’. For microscopy data, GraphPad Prism software v.10.1.1 was used to perform statistical analyses, using the Mann–Whitney *U* test or unpaired, two-tailed *t*-test. Values are presented as mean ± s.d. Statistical differences are represented as follows: *P < 0.05, **P < 0.01, ***P < 0.001, ****P < 0.0001.

### Reporting summary

Further information on research design is available in the Nature Portfolio Reporting Summary linked to this article.

## Online content

Any methods, additional references, Nature Portfolio reporting summaries, source data, extended data, supplementary information, acknowledgements, peer review information; details of author contributions and competing interests; and statements of data and code availability are available at https://doi.org/10.1038/s41587-025-02851-1.

## Supplementary information

Reporting Summary

Supplementary Video 1 Live time-lapse imaging of mouse preimplantation development. Mouse embryos were electroporated at the 8-cell stage with H2B-mCherry mRNA, resulting in mosaic labeling. The video displays maximum projections of live-cell time-lapse imaging of mouse preimplantation embryos expressing nuclear H2B-mCherry.

Supplementary Video 2 Live time-lapse imaging of human blastocyst development labeled with H2B-mCherry. Human blastocysts were electroporated with H2B-mCherry mRNA, resulting in mosaic labeling (not all cells labeled). Fluorescent images were taken every 15 min for 46 h.

Supplementary Video 3 Examples of lagging chromosomes observed in mouse and human blastocyst cells. The positions of the cells within the embryos are shown at lower magnification, followed by magnified views of the cells of interest undergoing division.

Supplementary Video 4 Examples of mitotic slippage observed in mouse and human blastocyst cells. Cells undergoing slippage are marked with a red arrow, while those undergoing normal division are marked with a green arrow.

Supplementary Video 5 Examples of normal and multipolar cell division observed in human blastocyst cells.

Supplementary Video 6 Examples of micronuclei inheritance observed in mouse and human blastocyst cells. Cells with micronuclei are marked with a red arrow, and micronuclei themselves are marked by arrowheads.

Supplementary Video 7 Fate of trophectoderm cells in human embryo 1. The video displays segmentation of human embryo nuclei labeled with H2B-mCherry and complete lineage tree from the early to late blastocyst stage, as well as the number of observed nuclei across time.

Supplementary Video 8 Fate of TE cells in human embryo 2. The video displays segmentation of human embryo nuclei labeled with H2B-mCherry and complete lineage tree from the early to late blastocyst stage, as well as the number of observed nuclei across time.

Supplementary Video 9 Fate of TE cells in human embryo 3. The video displays segmentation of human embryo nuclei labeled with H2B-mCherry and complete lineage tree from the early to late blastocyst stage, as well as the number of observed nuclei across time.

Supplementary Video 10 Video of transient-outer trophectoderm cell moving to inside position in a human embryo. The video displays a 3D view of a mother cell dividing into two daughter cells, with one remaining outside and the other moving to an inside position. The final position of the daughter cells is shown. The nucleus of the mother cell and the daughter cells are marked with green spots. Arrows mark the final positions of the daughter cells.

Supplementary Video 11 The video displays segmentation of transient-outer cell moving inwards. The transient outer daughter cell is marked with red color.

## Source data

Source Data Fig. 1 Statistical source data.

Source Data Fig. 2 Statistical source data.

Source Data Fig. 3 Statistical source data.

Source Data Fig. 4 Statistical source data.

Source Data Fig. 5 Statistical source data.

Source Data Fig. 6 Statistical source data.

Source Data Extended Data Fig. 2 Statistical source data.

Source Data Extended Data Fig. 5 Statistical source data.

Source Data Extended Data Fig. 7 Statistical source data.

Source Data Extended Data Fig. 9 Statistical source data.

## Data Availability

Raw light-sheet imaging data are available via Zenodo at https://doi.org/10.5281/zenodo.16996800 (ref. ^67^) and https://doi.org/10.5281/zenodo.16994339 (ref. ^68^). Images of human embryos are also accessible at EMBL-EBI BioImage Archive: S-BIAD2325. All other data supporting the findings of this study are available from the corresponding author. Source data are provided with this paper.

## Code availability

Semi-automated 3D nuclear segmentation and tracking was performed using (1) a custom version (https://github.com/akarsa/star-3d) of the StarDist-3D network tailored to our data, and (2) regularized Gaussian mixture optimal transport (GMMOT). Both are available for download (https://github.com/akarsa/star-3d, https://github.com/akarsa/optimal3dtracks). The segmented nuclei were tracked using regularized GMMOT (https://github.com/akarsa/optimal3dtracks, https://github.com/judelo/gmmot) to calculate transition probabilities between positions at consecutive time points.
