# Supplementary material for: Live imaging of late-stage preimplantation human embryos reveals de novo mitotic errors
Source: Nat Biotechnol. Author manuscript; Available in PMC 2026 Mar 24. (PMC7618924; doi:10.1038/s41587-025-02851-1)
Supplement: Reporting Summary [file EMS213036-supplement-Reporting_Summary.pdf]

nature portfolio

Corresponding author(s): Kathy K Niakan

Last updated by author(s): Aug 26, 2025

Reporting Summary

Nature Portfolio wishes to improve the reproducibility of the work that we publish. This form provides structure for consistency and transparency in reporting. For further information on Nature Portfolio policies, see our Editorial Policies and the Editorial Policy Checklist.

Statistics

For all statistical analyses, confirm that the following items are present in the figure legend, table legend, main text, or Methods section.

n/a	Confirmed	
☐	☒	The exact sample size (*n*) for each experimental group/condition, given as a discrete number and unit of measurement
☐	☒	A statement on whether measurements were taken from distinct samples or whether the same sample was measured repeatedly
☐	☒	The statistical test(s) used AND whether they are one- or two-sided<br>*Only common tests should be described solely by name; describe more complex techniques in the Methods section.*
☐	☒	A description of all covariates tested
☐	☒	A description of any assumptions or corrections, such as tests of normality and adjustment for multiple comparisons
☐	☒	A full description of the statistical parameters including central tendency (e.g. means) or other basic estimates (e.g. regression coefficient) AND variation (e.g. standard deviation) or associated estimates of uncertainty (e.g. confidence intervals)
☐	☒	For null hypothesis testing, the test statistic (e.g. *F*, *t*, *r*) with confidence intervals, effect sizes, degrees of freedom and *P* value noted<br>*Give P values as exact values whenever suitable.*
☒	☐	For Bayesian analysis, information on the choice of priors and Markov chain Monte Carlo settings
☒	☐	For hierarchical and complex designs, identification of the appropriate level for tests and full reporting of outcomes
☒	☐	Estimates of effect sizes (e.g. Cohen's *d*, Pearson's *r*), indicating how they were calculated

Our web collection on statistics for biologists contains articles on many of the points above.

Software and code

Policy information about availability of computer code

Data collection	Live imaging of mouse and human embryos was predominantly performed using a Viventis LS2-Live dual illumination and inverted detection microscope. Time-lapse images of embryos were captured every 15 minutes for up to 2 days at 37°C and 6% CO2. In 1 human embryo, Embryo 1 we used the Viventis LS1 microscope which has single illumination.<br>IMARIS software 9 (Bitplane, AG) and Fiji ImageJ open-source image processing package were used to reconstruct movies and analyse image data. Z spacing of 2 μm between slices and a 100 ms exposure time for each slice was performed. Laser intensity was minimized to obtain a reasonable signal-to-noise ratio from the raw data while minimizing phototoxicity. The reconstruction of movies was performed using IMARIS software (Bitplane, AG) and Fiji ImageJ open-source image processing package.
Data analysis	Statistical analysis was performed in Graphpad prism (8.4.3).<br>Semi-automated 3D nuclear segmentation and tracking was performed using 1) a custom version (https://github.com/akarsa/anisotropic_stardist_3d) of the StarDist-3D network tailored to our data, and 2) Optimal3dTracks, a regularized Gaussian mixture optimal transport bases method. Both softwares are available for download (https://github.com/akarsa/anisotropic_stardist_3d, https://github.com/akarsa/ cell_tracking_with_optimal_transport).<br>The segmented nuclei were tracked using regularized Gaussian mixture optimal transport (GMMOT) (https://github.com/akarsa/cell_tracking_with_optimal_transport, https://github.com/judelo/gmmot, to calculate transition probabilities between positions at consecutive time points. Using an in-house Python script, the automatically generated 3D segmentations and tracks were converted to a format readable by Fiji's TrackMate tool.<br>FIJI 2.3.0 was used for image analysis.

For manuscripts utilizing custom algorithms or software that are central to the research but not yet described in published literature, software must be made available to editors and reviewers. We strongly encourage code deposition in a community repository (e.g. GitHub). See the Nature Portfolio guidelines for submitting code & software for further information.

Data

Policy information about availability of data

All manuscripts must include a data availability statement. This statement should provide the following information, where applicable:

- Accession codes, unique identifiers, or web links for publicly available datasets
- A description of any restrictions on data availability
- For clinical datasets or third party data, please ensure that the statement adheres to our policy

Source data are provided with this paper. All other data supporting the findings of this study are available from the corresponding author. The primary microscopy data will be publicly available at publication.The data has been uploaded onto Zenodo and a URL is provided in the data availability statement (https://zenodo.org/records/16996801
https://zenodo.org/records/16994340)

Research involving human participants, their data, or biological material

Policy information about studies with human participants or human data. See also policy information about sex, gender (identity/presentation), and sexual orientation and race, ethnicity and racism.

Reporting on sex and gender	The embryos used in this study were not tested for sex. This is because the information was not relevant to our studies and performing a biopsy of the embryos to determine the sex chromosomes would have interfered with the study design for live embryo imaging and may have affected viability. The total number of embryos used is stated in the manuscript.
Reporting on race, ethnicity, or other socially relevant groupings	This is not applicable.
Population characteristics	All donations were provided pseudonymized at the point of transfer to the research project.
Recruitment	Embryos donated to this study were surplus to assisted reproduction treatment.
Ethics oversight	This study was approved by the UK Human Fertilisation and Embryology Authority (HFEA): research licence numbers R0162 and R0152 and independently reviewed by the Health Research Authority's Research Ethics Committee IRAS projects 308099, 252286 and 272218.

Note that full information on the approval of the study protocol must also be provided in the manuscript.

Field-specific reporting

Please select the one below that is the best fit for your research. If you are not sure, read the appropriate sections before making your selection.

☒ Life sciences ☐ Behavioural & social sciences ☐ Ecological, evolutionary & environmental sciences

For a reference copy of the document with all sections, see nature.com/documents/nr-reporting-summary-flat.pdf

Life sciences study design

All studies must disclose on these points even when the disclosure is negative.

Sample size	A total of 13 human embryos and 20 mouse embryos were analysed across multiple independent experiments. The number of cells analysed per embryo is reported in each figure or figure legend.
Data exclusions	No data were excluded from the study
Replication	Experiment were replicated at least three times and the data were reproducible in the different attempts of replication.
Randomization	There were no functional experiments or treatments performed therefore randomization is not relevant to this study.
Blinding	There were no functional experiments or treatments performed therefore blinding is not relevant to this study.

Reporting for specific materials, systems and methods

We require information from authors about some types of materials, experimental systems and methods used in many studies. Here, indicate whether each material, system or method listed is relevant to your study. If you are not sure if a list item applies to your research, read the appropriate section before selecting a response.

Materials & experimental systems

n/a	Involved in the study
☐	☒ Antibodies
☐	☒ Eukaryotic cell lines
☒	☐ Palaeontology and archaeology
☐	☒ Animals and other organisms
☒	☐ Clinical data
☒	☐ Dual use research of concern
☒	☐ Plants

Methods

n/a	Involved in the study
☒	☐ ChIP-seq
☒	☐ Flow cytometry
☒	☐ MRI-based neuroimaging

Antibodies

Antibodies used	Primary antibodies used were CDX2 (BioGenex; MU392A-UC) at dilution 1:50, NANOG (2B Scientific, REC-RCAB000lP) at dilution 1:100, NANOG (R&D, AF1997) at dilution 1:200, and GATA3 (Abcam, ab199428) at dilution 1:100. Secondary antibodies were Donkey anti-Mouse Alexa Fluor 488, 594 or 647; Donkey anti-Rabbit Alexa Fluor 488, 594, or 647; and Donkey anti-Goat Alexa Fluor 488, 594 or 647 (ThermoFisher).
Validation	Antibody validation was reported on the manufacturer's website.

Eukaryotic cell lines

Policy information about cell lines and Sex and Gender in Research

Cell line source(s)	HEK 293T cell line originally sourced from ATCC.
Authentication	The cell line used was not authenticated.
Mycoplasma contamination	Cell lines were tested for mycoplasma.
Commonly misidentified lines (See ICLAC register)	No commonly misidentified lines were use in these studies.

Animals and other research organisms

Policy information about studies involving animals; ARRIVE guidelines recommended for reporting animal research, and Sex and Gender in Research

Laboratory animals	Female mice, aged four to eight weeks (C57BL6 × CBA) F1, were superovulated by intraperitoneal injection of 5 IU of pregnant mare serum gonadotrophin (PMSG; Sigma-Aldrich) followed 48hours later with an intraperitoneal injection of 5 IU of human chorionic gonadotrophin (HCG; Sigma-Aldrich) and mating with eight weeks or older (C57BL6 × CBA) F1 males. The mice were maintained under a 12-hour light–dark cycle, ambient temperature 19/22°C, and humidity 45/65%. Zygotes were isolated from oviducts of plugged mice at 0.5 days post fertilisation in FHM medium (Merck; MR-122-D) under mineral oil (Origio; ART-4008-5P), and cumulus cells were removed using hyaluronidase (Sigma-Aldrich; H4272). Alternatively, CD1 female and male mice were time mated and blastocysts were collected 4 days post fertilisation by flushing uteri with FHM medium. Blastocysts were immediately fixed in 4% paraformaldehyde.
Wild animals	Not applicable.
Reporting on sex	Not applicable.
Field-collected samples	Not applicable.
Ethics oversight	All procedures involving animals were conducted in accordance with the UK Home Office Licence Number PP8826065.

Note that full information on the approval of the study protocol must also be provided in the manuscript.

Plants

Seed stocks

Report on the source of all seed stocks or other plant material used. If applicable, state the seed stock centre and catalogue number. If plant specimens were collected from the field, describe the collection location, date and sampling procedures.

Novel plant genotypes

Describe the methods by which all novel plant genotypes were produced. This includes those generated by transgenic approaches, gene editing, chemical/radiation-based mutagenesis and hybridization. For transgenic lines, describe the transformation method, the number of independent lines analyzed and the generation upon which experiments were performed. For gene-edited lines, describe the editor used, the endogenous sequence targeted for editing, the targeting guide RNA sequence (if applicable) and how the editor was applied.

Authentication

Describe any authentication procedures for each seed stock used or novel genotype generated. Describe any experiments used to assess the effect of a mutation and, where applicable, how potential secondary effects (e.g. second site T-DNA insertions, mosiacism, off-target gene editing) were examined.
